# Apoptotic exosome-like vesicles regulate endothelial gene expression, inflammatory signaling, and function through the NF-κB signaling pathway

**DOI:** 10.1038/s41598-020-69548-0

**Published:** 2020-07-28

**Authors:** Francis Migneault, Mélanie Dieudé, Julie Turgeon, Déborah Beillevaire, Marie-Pierre Hardy, Alexandre Brodeur, Nicolas Thibodeau, Claude Perreault, Marie-Josée Hébert

**Affiliations:** 1 Research Centre, Centre Hospitalier de l’Université de Montréal (CRCHUM), Tour Viger - R12.414, 900 rue St-Denis, Montreal, QC H2X 0A9 Canada; 2 Université de Montréal, Montreal, QC Canada; 3 Institute for Research in Immunology and Cancer (IRIC), Montreal, QC Canada; 4 Canadian Donation and Transplantation Research Program, Edmonton, AB Canada

**Keywords:** Apoptosis, Extracellular signalling molecules

## Abstract

Persistent endothelial injury promotes maladaptive responses by favoring the release of factors leading to perturbation in vascular homeostasis and tissue architecture. Caspase-3 dependent death of microvascular endothelial cells leads to the release of unique apoptotic exosome-like vesicles (ApoExo). Here, we evaluate the impact of ApoExo on endothelial gene expression and function in the context of a pro-apoptotic stimulus. Endothelial cells exposed to ApoExo differentially express genes involved in cell death, inflammation, differentiation, and cell movement. Endothelial cells exposed to ApoExo showed inhibition of apoptosis, improved wound closure along with reduced angiogenic activity and reduced expression of endothelial markers consistent with the first phase of endothelial-to-mesenchymal transition (endoMT). ApoExo interaction with endothelial cells also led to NF-κB activation. NF-κB is known to participate in endothelial dysfunction in numerous diseases. Silencing NF-κB reversed the anti-apoptotic effect and the pro-migratory state and prevented angiostatic properties and CD31 downregulation in endothelial cells exposed to ApoExo. This study identifies vascular injury-derived extracellular vesicles (ApoExo) as novel drivers of NF-κB activation in endothelial cells and demonstrates the pivotal role of this signaling pathway in coordinating ApoExo-induced functional changes in endothelial cells. Hence, targeting ApoExo-mediated NF-κB activation in endothelial cells opens new avenues to prevent endothelial dysfunction.

## Introduction

Endothelial injury and apoptosis are pivotal to a flurry of disease states ranging from atherosclerosis to ischemia–reperfusion injury, rejection of transplanted organs and chronic renal failure^1–4^. In renal transplantation, the severity of ischemia–reperfusion (IR) injury at the time of transplantation is an important predictor of long-term renal failure. Our group recently showed that injury and caspase-3 dependent death of microvascular endothelial cells is pivotal to progressive renal dysfunction following IR^3^. Persistent endothelial apoptosis fuels maladaptive responses by favoring the release of fibrogenic factors leading to perturbation in vascular homeostasis and tissue architecture^5–12^. Recently, we identified a novel type of extracellular vesicles (EVs), apoptotic exosome-like vesicles (ApoExo), as an additional component of the secretome of apoptotic endothelial cells produced downstream of caspase-3 activation and of potential importance in perturbing vascular homeostasis^13^.

EVs are classically categorized into three groups: exosomes, microvesicles and apoptotic bodies. They are encircled by a lipid bilayer membrane and contain molecular constituents from the parental cells including proteins and RNA that are protected from enzymatic degradation^14^. EVs are increasingly considered as intercellular communication devices, providing important signals to neighboring cells, but also distant cells because of their capacity to reach the bloodstream, evade degradation and circulate throughout the body. EVs can modulate gene expression and signaling pathways through their interaction with specific membrane receptors^15^ or upon the transfer of functionally active molecules into target cells following internalization^16,17^.

ApoExo represent a new class of EVs. They are distinct from classical apoptotic bodies by their size ranging from 30 to 100 nm, their protein content, and their enzymatic activity^12,13,18,19^. They are characterized by the expression of an active 20S proteasome core complex regulating their immunogenic activity. Indeed, ApoExo, contrary to apoptotic bodies, induce a pro-inflammatory response in naïve and transplanted mice and fuel the production of autoantibodies^13,20^. Inhibiting the proteasome activity of ApoExo with bortezomib largely reduces their immunogenic activity. Recent work by our group also shows that the RNA content of endothelial-derived ApoExo differs in quantity and quality from that of classical endothelial-derived exosomes^21^. Taken together, these recent findings support the notion that dying cells produce specific types of EVs that are aimed at alerting the immune system and triggering a pro-inflammatory response.

Endothelial cells are central to the maintenance of vessel wall homeostasis. They regulate vascular tone and, in their normal state, prevent inflammation and thrombosis. Recent studies suggest that EVs can behave as regulators of endothelial functions. Serum-derived exosomes can impair endothelial-dependent vasodilation by decreasing nitric oxide bioavailability through the inhibition of endothelial nitric oxide synthase expression^22–24^. Platelet-derived exosomes may also contribute to endothelial dysfunction by disturbing the redox metabolism^25,26^. It also has been described that EVs can induce^23,27^ or resolve^28^ endothelial activation through modulation of ICAM-1 expression, thereby modulating vascular permeability and rolling/adhesion of inflammatory cells. The impact of ApoExo on endothelial homeostasis and function remains uncharacterized.

In the present work, we aim to characterize the impact of ApoExo on endothelial gene expression and function. Using transcriptomic analysis and functional experiments, we show that ApoExo activate the NF-κB pathway in endothelial cells leading to important changes in their differentiation state and their migratory and angiogenic activities.

## Results

### Apoptotic exosome-like vesicles impact endothelial gene expression

Endothelial cells were serum-starved to induce apoptosis, as described previously^13^. This leads to classical features of apoptosis such as chromatin condensation in the absence of membrane permeabilization and cleavage of caspase-3 (Fig. S1). Caspase-3 activation in endothelial cells prompts the release of ApoExo and apoptotic bodies, both of which can, in turn, interact with neighboring endothelial cells and potentially impact their functions^13,18^. We used sequential centrifugation to purify apoptotic bodies and ApoExo produced by serum-starved endothelial cells and quantified the two populations (Fig. S2A–D). Electron microscopy performed on both fractions isolated by sequential centrifugation showed distinct ultrastructural characteristics as previously described^13^; apoptotic bodies being within the micron range whereas the size of ApoExo was within 30–100 nm (Fig. S2E). Immunoblot analysis comparing the protein profiles of apoptotic bodies and ApoExo showed enrichment in the exosome markers synthenin, and TCTP, but not the tetraspanin CD63. GM130 was used as purity control and was not expressed in ApoExo. Moreover, perlecan/LG3, 20S proteasome, and LAMP2, previously characterized as markers of ApoExo, were found to be expressed by ApoExo but not by apoptotic bodies (Fig. S2F)^13,21^, confirming the difference between both types of extracellular vesicles (EV).

**Figure 1:**
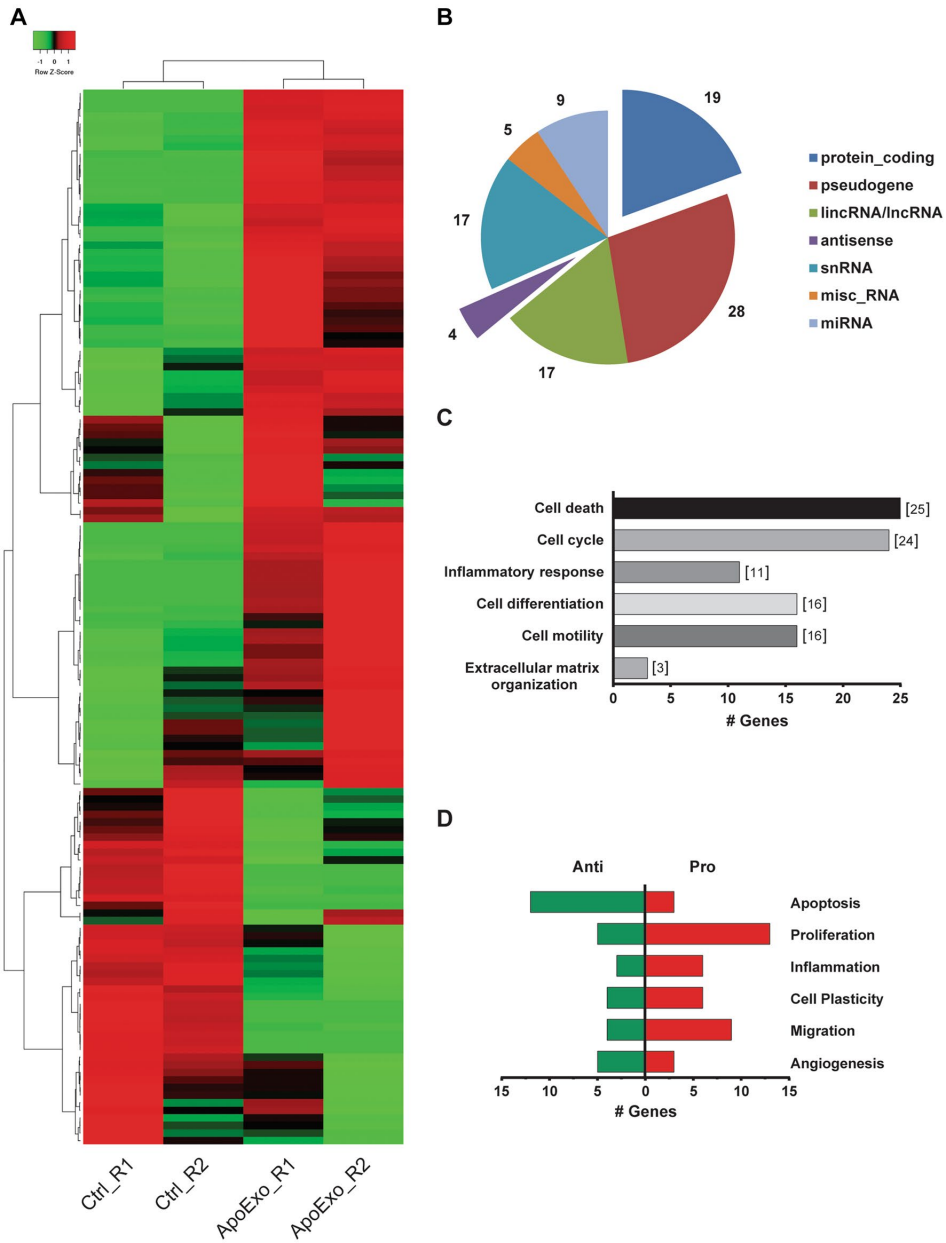
Apoptotic exosome-like vesicles modulate gene expression of endothelial cells. (**A**) Heatmap of the 139 differentially expressed genes between the control and the apoptotic exosome-like vesicles-treated groups. Apoptotic exosome-like vesicles treatment increased the expression of 92 genes and a decrease of 47 genes. (**B**) Pie chart representation of the distribution of differentially expressed genes expressed as the percentage of each transcript biotype. (**C**) Gene ontology analysis of biological processes of differentially expressed antisense and protein-coding genes. (**D**) Characterization of differentially expressed genes according to endothelial functions. n = 2 for each condition.

We then used RNA-seq analysis in endothelial cells exposed to ApoExo or vehicle to identify trends in gene expression that could hint to the functional impact of ApoExo on endothelial function. 139 genes were found to be differentially expressed between endothelial cells exposed to ApoExo compared to vehicle-treated cells (Fig. 1A and Table S1). The distribution of these genes was as follows: pseudogenes (28%), protein-coding genes (19%), snRNAs (17%), LincRNAs (17%) and others (19%) (Fig. 1B). Protein-coding and antisense RNAs were further analyzed, again to identify biological processes regulated by ApoExo. The antisense targets and protein-coding genes were categorized according to their Gene Ontology annotation, Ingenuity Pathway Analysis, and the literature. Genes modulated by ApoExo belong to the following functional categories: cell death, cell cycle, inflammation, differentiation, motility and extracellular matrix organization (Fig. 1C,D).

### Apoptotic exosome-like vesicles regulate endothelial survival, migration, angiogenesis, and differentiation

**Figure 2:**
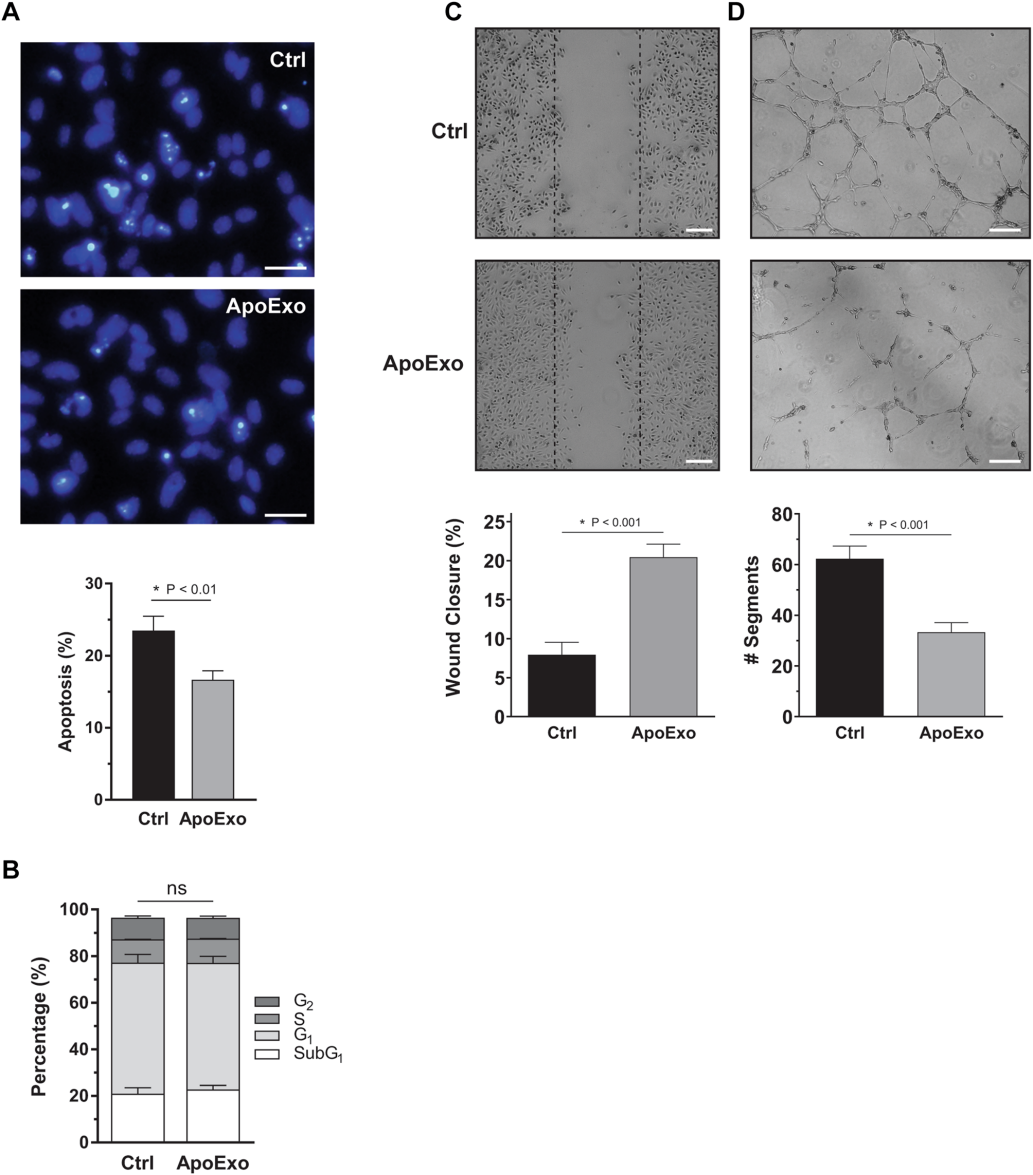
Apoptotic exosome-like vesicles inhibit apoptosis, improve wound closure, and decrease angiogenic activity in endothelial cells. (**A**) Apoptotic exosome-like vesicles (ApoExo) increased cell survival under serum starvation. Evaluation by Hoescht and Propidium iodide (HO/PI) staining of apoptotic or necrotic cells in serum-starved endothelial cells exposed for 4 h to the vehicle (Ctrl) or apoptotic exosome-like vesicles (ApoExo) (Scale bar: 25 µm). HO/PI experiments expressed as the percentage of apoptosis ± SEM. n = 11 for each condition. (**B**) ApoExo did not affect the cell cycle. Cell cycle analysis by PI staining of endothelial cells under serum starvation exposed for 12 h to the vehicle (Ctrl) or apoptotic exosome-like vesicles (ApoExo). Cell cycle experiments expressed as the percentage of cells under each cycle stage ± SEM. n = 3 for each condition. (**C**) ApoExo increased wound closure. Serum-starved endothelial cells were mechanically injured and exposed to the vehicle (Ctrl) or apoptotic exosome-like vesicles (ApoExo). Wound closure was followed over a 12 h period (Scale bar: 200 µm). Wound healing assay expressed as the percentage of wound closure ± SEM. n ≥ 6 for each condition. Representative pictures at 12 h post-injury are presented. (**D**) ApoExo decreased tubules formation of endothelial cells. Serum-starved endothelial cells were exposed to the vehicle (Ctrl) or apoptotic exosome-like vesicles (ApoExo) and capillary-like structures were quantified following a 7 h treatment on extracellular matrix (Scale bar: 200 µm). Capillaries density is depicted as the number of segments per field ± SEM and representative pictures of tubule formations are presented. n ≥ 3 for each condition. *P* values obtained by unpaired t-test.

We then evaluated whether modulated gene expression identified by RNA-seq translated into alterations in endothelial functions. RNA-seq predicted the acquisition of an anti-apoptotic phenotype in endothelial cells exposed to ApoExo. To confirm these results, endothelial cells were exposed to serum-free medium, a classical pro-apoptotic stimulus, in presence of ApoExo or vehicle. Lower apoptosis levels were found in endothelial cells exposed to ApoExo (Fig. 2A). However, while RNA-seq suggested a pro-proliferative response in endothelial cells exposed to ApoExo, this did not translate into enhanced endothelial proliferation. Indeed, cell cycle analysis was comparable in endothelial cells exposed to ApoExo or vehicle and more specifically there was no difference in the number of cells entering the G2/S phase (Fig. 2B). As increased migration was predicted by RNA-seq, endothelial migration was assessed with a wound-healing assay of endothelial monolayers exposed to ApoExo or vehicle. ApoExo did promote endothelial migration (Fig. 2C). To rule out any possible variation caused by the difference in the width of the scratches, we also measured the initial open wound area. Open wound areas were consistent between the experimental conditions showing the specificity of ApoExo in the modulation of migration (Fig. S3). Moreover, since ApoExo did not induce the proliferation of endothelial cells, the increased number of cells migrating within the denuded area could not have been caused by increased proliferation.

As RNA-seq suggested reduced expression of pro-angiogenic genes in the presence of ApoExo, we then evaluated the functional impact of ApoExo on angiogenesis in vitro. Endothelial cells grown on Matrigel were exposed to ApoExo or control for 7 h. Tube formation was significantly reduced in the presence of ApoExo (Fig. 2D). The total number of segments formed, the number of nodes and junctions and the segment lengths were also significantly reduced in the presence of ApoExo (Fig. S4). Differential ultracentrifugation can result in the presence of protein aggregates in EV preparations. To evaluate the relative contribution of membrane-bound vesicles (ApoExo and apoptotic bodies) vs protein aggregates on migration and angiogenic activity, we used a number of biological and technical controls. Neither apoptotic bodies, exosome-like EVs secreted from healthy endothelial cells nor post-200,000×*g* supernatant modulated endothelial functions in ways similar to ApoExo (Fig. S5A,B). Also, treatment of ApoExo pellets with Triton X-100 abrogated their pro-migration activity, demonstrating that membrane-bound vesicles rather than protein aggregates are promoting migration (Fig. S5A).

**Figure 3:**
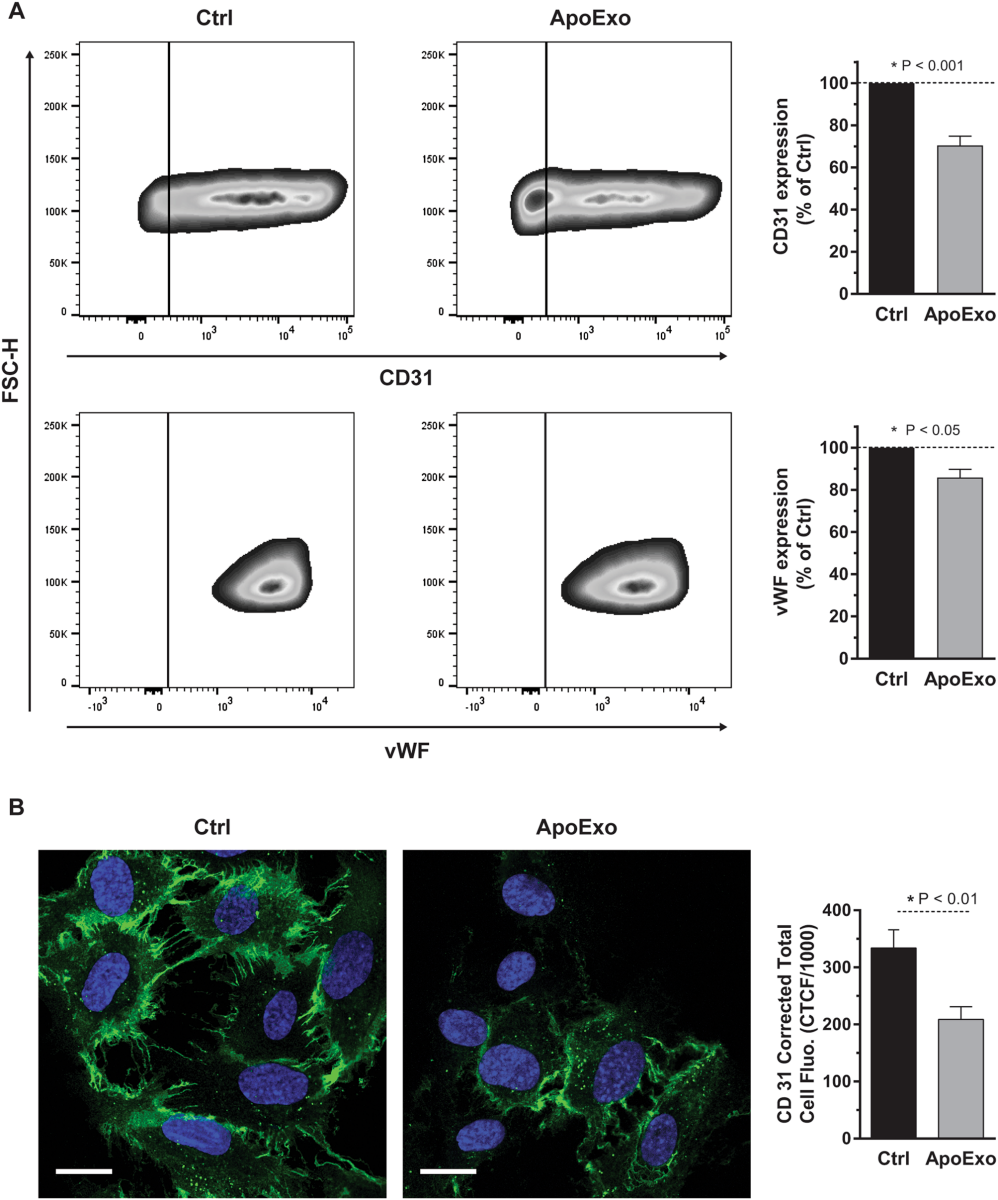
Apoptotic exosome-like vesicles decrease the expression of endothelial markers in endothelial cells. Apoptotic exosome-like vesicles (ApoExo) decreased CD31 and vWF expressions in endothelial cells. (**A**) CD31 and vWF expression by flow cytometry analysis in serum-starved endothelial cells exposed for 24 h to the vehicle (Ctrl) or apoptotic exosome-like vesicles (ApoExo). Flow cytometry experiments expressed as the percentage of vehicle-treated cells median fluorescence intensity (50,000 events/sample) ± SEM (right). Representative gates of CD31 and vWF expression are depicted (left). n ≥ 5 for each condition. *P* values obtained by a one-sample *t*-test. (**B**) ApoExo decreased CD31 expression in endothelial cells by confocal microscopy. (Scale bar: 20 µm). Semi-quantitative measurement of CD31 expression depicted as corrected total cell fluorescence (CTCF) ± SEM (right). Representative pictures of CD31 expression are presented (left). CTCF represents the integrated density minus the area of selected cell multiplied by the mean fluorescence of background reading. n = 3 for each condition. *P* value obtained by unpaired t-test.

Increased migration along with decreased angiogenic properties suggest the potential for endothelial dedifferentiation and the acquisition of a mesenchymal phenotype. RNA-seq suggesting enhanced expression of genes favoring endothelial plasticity also pointed in this direction. To evaluate this possibility, we evaluated the impact of ApoExo on the expression of two endothelial markers CD31 and von Willebrand Factor (vWF). Both markers showed rapid downregulation in the early phases of endothelial to mesenchymal transition^29,30^. Both CD31 and vWF (Fig. 3A) were downregulated by ApoExo as measured by flow cytometry. Confocal microscopy confirmed decreased expression of CD31 in endothelial cells exposed to ApoExo (Fig. 3B). We then evaluated whether the expression of mesenchymal markers such as α-smooth muscle actin (αSMA) and S100 calcium-binding protein A4 (S100A4) increased upon exposure to ApoExo. Endothelial cells exposed to ApoExo did not modulate their expression of αSMA or S100A4 when compared to endothelial cells exposed to vehicle (Fig. S6). Altogether, these results confirm that ApoExo not only change the gene expression pattern of endothelial cells, but also influence key functions leading to enhanced survival and migration but reduced angiogenesis. Although ApoExo favor endothelial dedifferentiation with reduced expression of CD31 and vWF, they do not induce complete mesenchymal transition as demonstrated by unaltered levels of αSMA and S100A4.

### NF-κB activation is central to the modulation of endothelial function induced by apoptotic exosome-like vesicles

**Figure 4:**
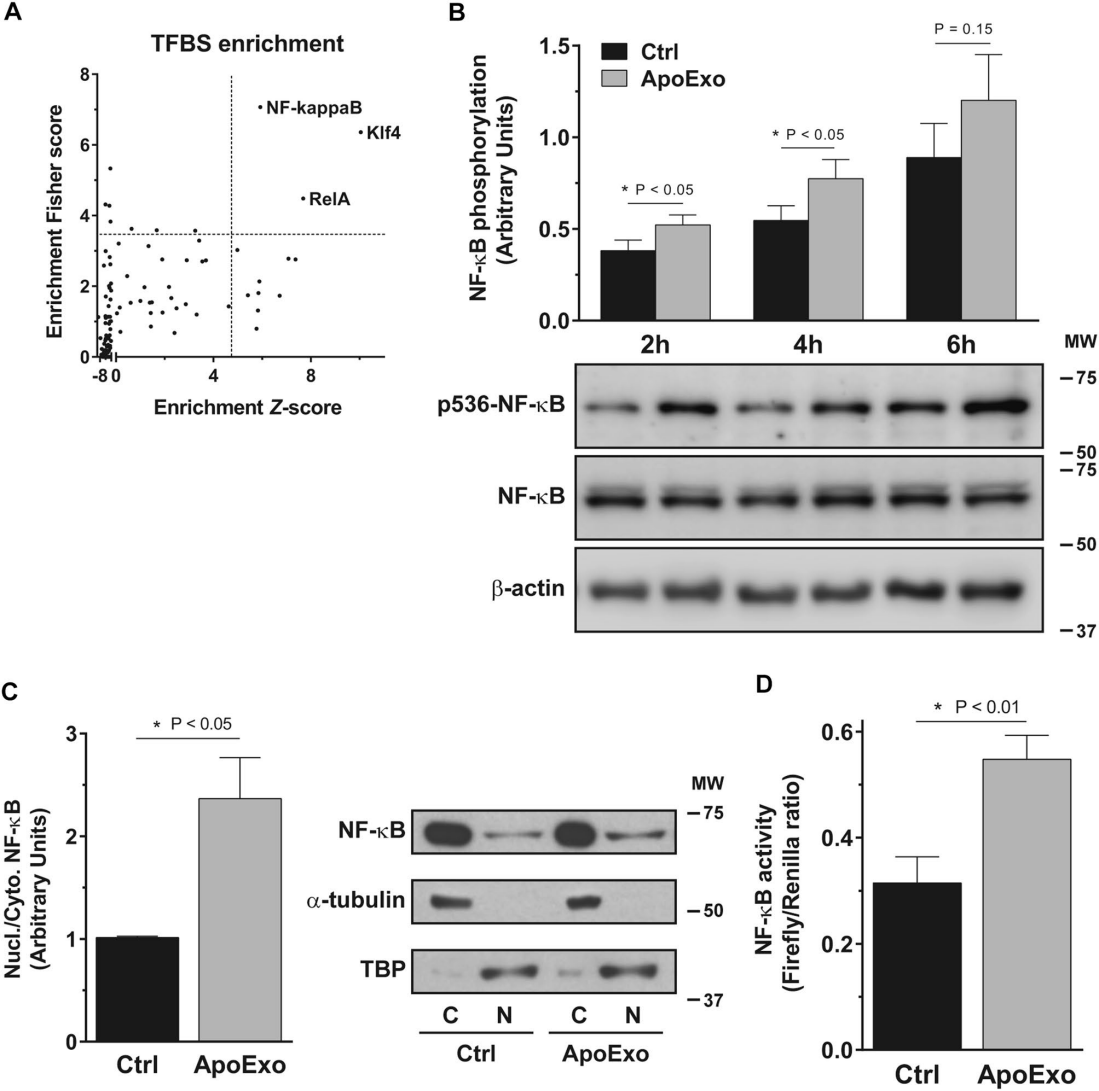
Apoptotic exosome-like vesicles trigger the activation of the NF-κB signaling pathway. (**A**) NF-κB transcription factors family binding sites are enriched on the promoter of differentially expressed genes. Differentially expressed protein-coding genes from RNA-sequencing analysis were processed through oPOSSUM 3.0 using single-site analysis. The transcription factor binding sites (TFBS) were then classified according to their Fisher score and Z-score. TFBS were considered overrepresented when both scores > mean score + 1.5 fold standard deviation (dot lines). (**B**) Apoptotic exosome-like vesicles (ApoExo) increase NF-κB phosphorylation in endothelial cells. Serum-starved endothelial cells were exposed to the vehicle (Ctrl) or apoptotic exosome-like vesicles (ApoExo) and phosphorylation of NF-κB at Ser536 (p536-NF-κB) was followed from 2 to 6 h. NF-κB phosphorylation was quantified by the phospho-to-total ratio for each time point and expressed as arbitrary units ± SEM. n ≥ 11 for each condition. Representative immunoblots cropped from the same gel are presented. (**C**) ApoExo trigger NF-κB translocation in endothelial cells. Serum-starved endothelial cells were exposed to the vehicle (Ctrl) or apoptotic exosome-like vesicles (ApoExo) for 4 h and NF-κB (RelA) expression was assessed. NF-κB translocation was quantified by the nuclear (N) to cytoplasmic (C) ratio and expressed as arbitrary units ± SEM. n = 6 for each condition. Representative immunoblots cropped from the same gel are presented. (**D**) ApoExo increase NF-κB activity in endothelial cells. Serum-starved endothelial cells were exposed to the vehicle (Ctrl) or apoptotic exosome-like vesicles (ApoExo) for 4 h and NF-κB activity was expressed as Firefly/Renilla luciferases ratio ± SEM. n = 6 for each condition. *P* values obtained by a one-tailed unpaired t-test. Full-length blots are presented in Supplementary Fig. 12.

To characterize the molecular pathways that trigger the phenotypic changes induced by ApoExo we went back to the list of differentially expressed protein-coding genes identified by RNA-seq and we used single-site analysis using oPOSSUM 3.0^31^ to focus on over-represented conserved transcription factor binding sites (TFBS). Single site analysis identified three overrepresented transcription factors binding sites: two from the NF-κB family (NF-kappaB and RelA) and the KLF4 transcription factor (Fig. 4A). To investigate further the importance of NF-κB in the endothelial response to ApoExo, we assessed the phosphorylation of Ser536 on the RelA subunit since this phosphorylation site is known to enhance the activation of the transcription factor^32^. Immunodetection revealed enhanced NF-κB phosphorylation at Ser536 in endothelial cells exposed specifically to ApoExo (Fig. 4B and S5C). Second, we measured the nuclear-to-cytoplasm ratio of NF-κB since NF-κB must translocate to the nucleus to modulate the transcription of target genes. As expected, ApoExo increased NF-κB nuclear translocation (Fig. 4C). Finally, we used a luciferase reporter assay to assess the nuclear activity of NF-κB and confirmed increased nuclear activity in endothelial cells exposed to ApoExo (Fig. 4D).

**Figure 5:**
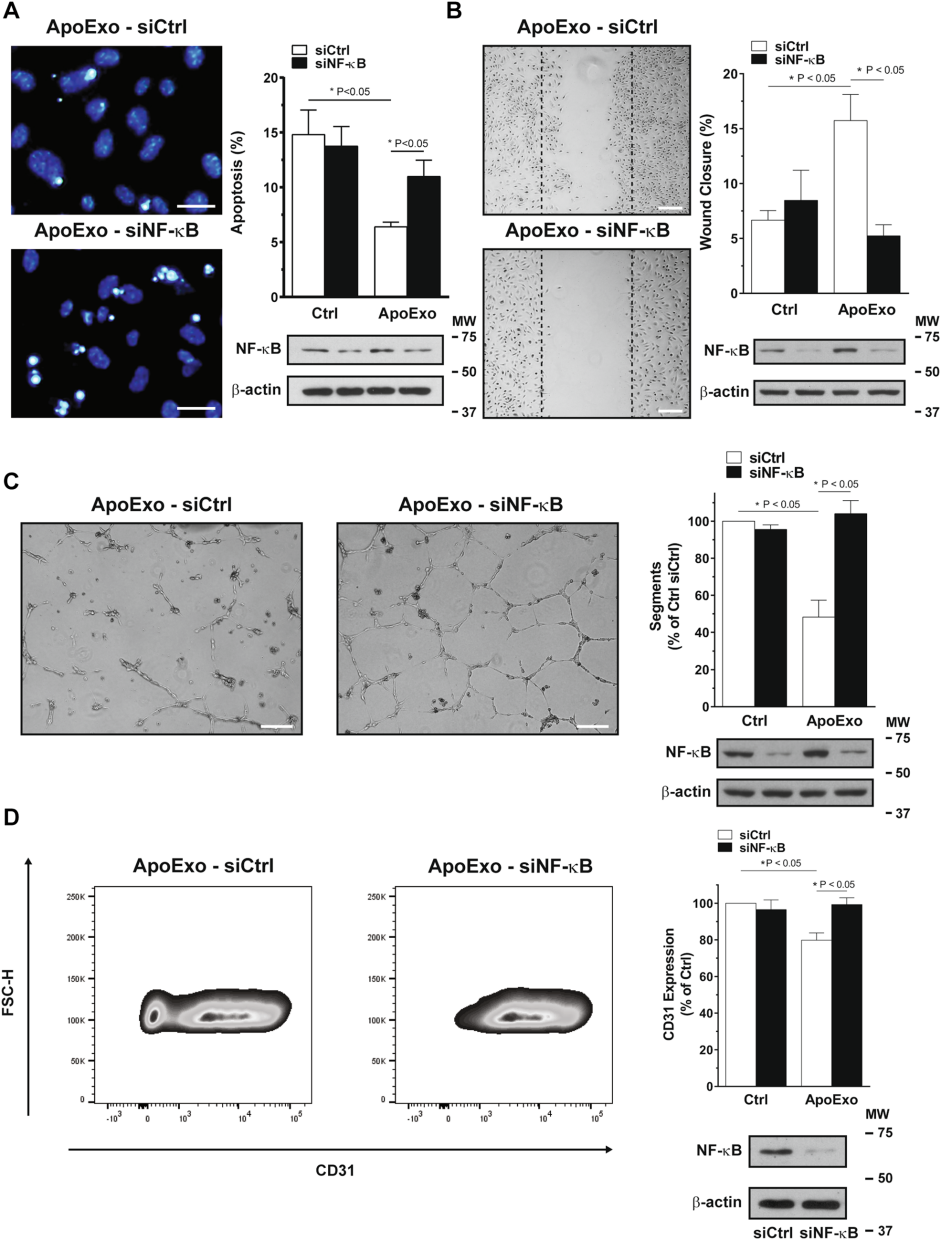
Apoptotic exosome-like vesicles mediate endothelial dedifferentiation through NF-κB activation. (**A**) NF-κB knock-down impaired the anti-apoptotic effect induced by apoptotic exosome-like vesicles (ApoExo). Serum-starved endothelial cells transfected with Ctrl or NF-κB siRNA 90 nM were exposed for 4 h to the vehicle (Ctrl) or apoptotic exosome-like vesicles (ApoExo). Apoptotic or necrotic cells were assessed by Hoescht and Propidium iodide (HO/PI) staining (Scale bar: 25 µm). HO/PI experiments expressed as the percentage of apoptosis ± SEM. n = 3 for each condition. Representative pictures and cropped immunoblots from the same gel of NF-κB knock-down at 4 h post-starvation are presented. P values were obtained by one-way ANOVA and Bonferroni's post hoc test. (**B**) NF-κB inhibition repressed wound closure induced by apoptotic exosome-like vesicles (ApoExo). Serum-starved endothelial cells transfected with Ctrl or NF-κB siRNA 90 nM were mechanically injured and exposed to the vehicle (Ctrl) or apoptotic exosome-like vesicles (ApoExo). Wound closure was followed over a 12 h period (Scale bar: 200 µm). Wound healing assay expressed as the percentage of wound closure ± SEM. n = 3 for each condition. Representative pictures and cropped immunoblots from the same gel of NF-κB knock-down at 12 h post-injury are presented. *P* values were obtained by one-way ANOVA and Bonferroni's post hoc test. (**C**) NF-κB knock-down restore tubules formation decreased by ApoExo. Serum-starved endothelial cells transfected with Ctrl or NF-κB siRNA 90 nM were exposed to the vehicle (Ctrl) or apoptotic exosome-like vesicles (ApoExo) and capillary-like structures were quantified following a 7 h treatment on extracellular matrix (Scale bar: 200 µm). Capillaries density is depicted as the number of segments per field ± SEM and representative pictures of tubules formation and cropped immunoblots from the same gel of NF-κB knock-down are presented. n = 4 for each condition. P values were obtained by one-way ANOVA and Bonferroni's post hoc test. (**D**) NF-κB knock-down reestablishes CD31 expression inhibited by apoptotic exosome-like-vesicles. Serum-starved endothelial cells transfected with Ctrl or NF-κB siRNA 90 nM were exposed for 24 h to the vehicle (Ctrl) or apoptotic exosome-like vesicles (ApoExo) and CD31 expression was analyzed by flow cytometry. Flow cytometry graphs represent CD31 expression as the percentage of vehicle-treated cells median fluorescence intensity (50,000 events/sample) ± SEM. Representative gates of CD31 expression and cropped immunoblots from the same gel of NF-κB knock-down are depicted. n = 5 for each condition. *P* values were obtained by the Kruskal–Wallis test and Dunn’s post hoc test. Full-length blots are presented in Supplementary Fig. 13.

Next, we used NF-κB inhibition using siRNA to assess the importance of NF-κB activation in the modulation of endothelial functions by ApoExo. Inhibition of NF-κB signaling compromised the anti-apoptotic effect mediated by ApoExo (Fig. 5A). NF-κB silencing and pharmacological inhibition both impaired the pro-migratory phenotype induced by ApoExo (Fig. 5B and S7). We then evaluated the impact of NF-κB knock-down using siRNA on the formation of tubular-like networks of endothelial cells grown on Matrigel. NF-κB silencing prevented the anti-angiogenic activity of ApoExo. All angiogenic parameters (number of segments, nodes, junctions, and segment length) were restored by NF-κB silencing, demonstrating a broad inhibitory activity (Fig. 5C and S8). Finally, to assess the impact of NF-κB on ApoExo-induced endothelial dedifferentiation, we measured the expression of CD31 in ApoExo-treated endothelial cells silenced for NF-κB. NF-κB siRNA, but not control siRNA, re-established CD31 expression in endothelial cells exposed to ApoExo (Fig. 5D). Finally, we confirmed that NF-κB activation induced by ApoExo pellets was dependent on the presence of membrane-bound structures and not protein aggregates as treatment of ApoExo pellets with Triton X-100 abrogated their capacity to induce NF-κB activation (Fig. S5C). Collectively, these results demonstrate a pivotal role for NF-κB activation in controlling ApoExo-induced functional changes in endothelial cells.

## Discussion

Apoptotic cells release a wide array of EVs that in turn regulate immune functions and tissue repair in a variety of ways. Recently we showed that apoptotic cells release smaller EVs that share some but not all features of exosomes. Apoptotic exosome-like vesicles (ApoExo) appear to lack at least some classic exosome markers such as tetraspanins (CD9, CD63, and CD81) (Fig. S2)^13^, but express other markers such as perlecan/LG3 and the 20S proteasome complex^13,33^. Proteasome complex has been described in circulating EVs following ischemia–reperfusion injury or in association with autoimmune diseases^34,35^, or lung allograft rejection^36^. The present study identifies ApoExo as important regulators of endothelial gene expression, inflammatory signaling, and function. Using transcriptomic and functional approaches, we showed that ApoExo mediate a pro-survival, pro-migratory and anti-angiogenic phenotype in endothelial cells consistent with endothelial dedifferentiation of potential importance in vascular response to injury. Moreover, we identified activation of the transcription factor NF-κB as a central player in the regulation of ApoExo-induced effects on endothelial function.

Firstly, we sought to characterize the potential impact of ApoExo on endothelial gene expression as a means of hinting to potential changes in endothelial function. To this end, we employed a transcriptomic approach to identify the modulation of gene expression induced by ApoExo. We used RT-qPCR in a subset of genes to confirm the correlation between the two methods (Fig. S9). ApoExo interaction with endothelial cells led to rapid changes in their gene expression. The majority of differentially expressed genes were long non-coding RNA (pseudogenes and lncRNA). This observation is in stark contrast with the known impact of classical exosomes on gene expression as the latter have been rather associated with the expression of microRNA and coding mRNA. Classical exosomes are principally known to modulate transcripts encoding proteins for cytokines, membrane receptors, transcription factors, and signaling pathways in different cell types such as beta-cells, keratinocytes, fibroblasts, and peripheral blood mononuclear cells^37–40^. Whether non-coding RNAs expressed within ApoExo modulate endothelial functions will be the scope of future studies. Nonetheless, several protein-coding mRNAs were found to be regulated upon exposure to ApoExo and pointed to potential functions in the regulation of endothelial differentiation, migration, and angiogenesis.

Endothelial cells exposed to ApoExo showed inhibition of apoptosis, improved wound closure along with reduced angiogenic activity and reduced expression of endothelial markers. These results were consistent with the transcriptomic analysis, except for the lack of pro-proliferative status. However, this latter finding is not surprising taking into consideration that, in our model, endothelial cells are placed in serum-free conditions, a classical inducer of quiescence and G0/G1 arrest^19,41^. ApoExo-induced opposite responses in migration and angiogenesis, although consistent with the transcriptomic analysis, represented a deviation from conventional wisdom. Angiogenesis is typically associated with increased endothelial migration capacity. Other types of EVs, such as classical exosomes have been reported to foster both migration as well as angiogenesis^42–47^. These results bring further support to the notion that ApoExo, although similar in size with classical exosomes, represent a distinct entity with a unique functional pattern on endothelial function. We have also previously shown that ApoExo are enriched in the C-terminal fragment of perlecan LG3, known to display angiostatic properties^48–50^. Reduced angiogenesis in the face of increased migration can also represent a clue to the initial stages of endothelial-to-mesenchymal transition, where cells lose endothelial characteristics to express mesenchymal markers and behavior. The downregulation of CD31 and vWF endothelial markers expression following ApoExo treatment support this hypothesis. Similar to the more extensively studied epithelial-to-mesenchymal transition (EMT), endoMT is characterized by the ability of endothelial cells to gain a mesenchymal-like phenotype, resulting in a high migratory potential, the loss of characteristic endothelial markers and the expression of mesenchymal markers such as S100A4 (FSP-1), α-SMA and vimentin^51,52^. Evidence suggests that endoMT is essential to the embryonic and adult vascular development^53–56^, but also in vascular remodeling leading to the formation of intimal thickening^57^. Our results suggest that ApoExo trigger endothelial dedifferentiation consistent with the first phase of endoMT without the transition to a mesenchymal state. Endothelial cells undergo endoMT by gradually losing the expression of endothelial markers while gaining mesenchymal markers over time. This process occurs over a period of several days^58–60^. The absence of the second phase of endoMT in our system could be explained by the use of serum starvation, which cannot be maintained in the long-term on endothelial cells because of massive cell death.

Having determined that ApoExo can trigger functional and phenotypic changes in endothelial cells, we then characterized the signaling pathways regulating endothelial dedifferentiation, angiostasis, and migration. We found NF-κB binding sites to be enriched in the promoter region of the differentially expressed genes detected by RNA-sequencing. Immunodetection and luciferase reporter assay confirmed the activation of NF-κB in endothelial cells exposed to ApoExo. We also demonstrated the critical role of NF-κB activation in the functional changes induced by ApoExo. NF-κB knock-down reversed the anti-apoptotic effect and the pro-migratory state, restored angiogenic properties and CD31 expression in endothelial cells exposed to ApoExo. NF-κB has been reported to play a crucial role in preventing heat stress-induced early apoptosis in HUVEC cells^61^. NF-κB is also known to regulate endothelial cell migration^62,63^ and endothelial wound healing under serum starvation^64^. Several studies also reported that NF-κB activation is associated with angiogenesis inhibition^65–67^. Collectively, these results point to a pivotal role for ApoExo-induced NF-kB activation in controlling downstream gene expression and function. These results are also consistent with the impact of NF-κB activation as an initiator of endothelial dedifferentiation and endothelial-to-mesenchymal transition^30,68^.

ApoExo are known to foster inflammation in naïve and transplanted mice in addition to promoting the production of autoantibodies through a proteasome-dependent mechanism^13,20^. However, inhibiting the proteasome activity of ApoExo did not alleviate the endothelial phenotype induced by ApoExo suggesting that mediators other than the proteasome are responsible for this effect (Fig. S10). LG3, another marker of ApoExo, has been reported to inhibit endothelial migration in a number of cell and animal systems^48,49^ and is therefore unlikely to mediate the impact of ApoExo on endothelial function. However, since LG3 is a C-terminal fragment of perlecan, other perlecan fragments or the native protein itself could be present in ApoExo and modulate endothelial responses. Depending on sites of degradation, perlecan can display angiogenic or angiostatic activities. The specific contribution of perlecan and its LG3 fragment on the functional modulation of endothelial cells by ApoExo will be the scope of future investigations. Our group also showed that ApoExo contain RNA suited to stimulate a large variety of toll-like receptors (TLRs) and RIG-I-like receptors (RLRs) known to activate the NF-kB pathway^21^. Several reports have also described the presence of DNA species in classical exosomes, which have the potential to activate DNA-sensor receptors in cells^69–71^. We showed that DNA was indeed detectable in ApoExo, but its low level and the absence of histones in these vesicles make its involvement in NF-κB activation unlikely (Fig. S11). Proteomic data of ApoExo also identified integrins (ITGA5 and ITGB1) known to trigger an intracellular signaling pathway of potential importance in NF-kB activation^13^. Characterizing the detailed molecular machinery linking ApoExo to NF-κB activation will be the scope of future studies.

Endothelial cells are the guardians of vascular homeostasis, they regulate leukocyte trafficking, coagulation, and vascular tone and different vascular beds display important functional differences and stress response. However, we determined previously that ApoExo are released not only by HUVECs, our model system but also by murine aortic endothelial cells as well as other cellular constituents of the vessel wall^13,20^. The present results suggest that the release of ApoExo by damaged endothelial cells can have important and broad repercussions on vascular functions due to their capacity to access the bloodstream, resist degradation and impact endothelial cells throughout the vascular tree. We showed previously that different models of ischemia–reperfusion injury favor the release of ApoExo within the circulation^13^. Here, we demonstrate that ApoExo interaction with endothelial cells leads to NF-κB activation and functional changes of importance in endothelial dysfunction. NF-κB activation is known to contribute to endothelial dysfunction and vascular remodeling in allograft vasculopathy^72,73^ and other disease states^74–77^. The present, by identifying ApoExo as novel drivers of NF-κB activation in endothelial cells, opens new avenues to prevent endothelial dysfunction.

Collectively, our results suggest that ApoExo, released by apoptotic endothelial cells can shape endothelial gene expression and functions in directions conducive to endothelial dysfunction and mesenchymal transition.

## Materials and methods

### Cell culture and reagents

Human umbilical vein endothelial cells (HUVEC) were purchased from Cell Applications, cultured in EGM-2MV complete medium (Lonza) or Medium 200 + LSGS (Gibco, Waltham, MA, USA) on a gelatin-coated surface and used at passage 4. To produce a conditioned medium containing ApoExo, cells were exposed to RPMI serum-free medium (Gibco) for 4 h to induce apoptosis in endothelial cells. Serum starvation is a classical inducer of apoptosis. We showed previously that serum starvation in endothelial cells increases chromatin condensation in the absence of cell membrane permeabilization along with caspase-3 activation^11–13^.

### Extracellular vesicle isolation

Conditioned serum-free media was fractioned by sequential centrifugation as previously described (Fig. S2)^13^. Briefly, it is consisted of first centrifugation at 1,200×*g* for 15 min at 4 °C to remove dead cells and debris. The supernatant was subjected to a 50,000×*g* centrifugation for 15 min at 4 °C to pellet apoptotic bodies and then final centrifugation at 200,000×*g* for 18 h at 4 °C to pellet ApoExo. Apoptotic bodies and ApoExo were purified from serum-free medium conditioned by 4.9 × 10^6^ HUVEC and resuspended in the initial volume of conditioned medium (25 mL) for the experimental procedures.

### Whole transcriptome sequencing (RNA-Seq)

Cells were grown on gelatin-coated glass coverslips until confluence and incubated with ApoExo or vehicle for 2 h. Total RNA was isolated from a 6-well by directly lysing the cells with TRIzol Reagent according to the manufacturer’s protocol (ThermoFisher) and then further purified using RNeasy columns (Qiagen, Germantown, MD, USA). The following steps were adapted from Hardy et al.^21^. Briefly, the presence of contamination with chemicals was assessed by Nanodrop using 260/280 and 260/230 ratios. Quantification of total RNA was made by QuBit (Applied Biosystems, Waltham, MA, USA) and 1,000 ng of total RNA was used for library preparation. Quality of total RNA was assessed with the BioAnalyzer Nano (Agilent, Santa Clara, CA, USA) and all samples had a RIN above 9. Library preparation was done with the KAPA mRNAseq stranded kit (KAPA, Cat no. KK8420). Ligation was made with 11 nM final concentration of Illumina index and 9 PCR cycles were required to amplify cDNA libraries. Libraries were quantified by QuBit and BioAnalyzer. All libraries were diluted to 10 nM and normalized by qPCR using the KAPA library quantification kit (KAPA; Cat no. KK4973). Libraries were pooled to equimolar concentration. Sequencing was performed with the Illumina Hiseq2000 using the Hiseq Reagent Kit v3 (200 cycles, paired-end) using 1.7 nM of the pooled library. Around 39–122 M paired-end PF reads were generated per sample. Sequencing adapters were masked from the end of both sets of 100 bp reads prior to the alignment using Trimmomatic version 0.35. Data were aligned to the reference human genome version GRCh38 (gene annotation from Gencode version 24) using STAR alignment version 2.5.1b. RNA-Seq data have been deposited in GEO archives (https://www.ncbi.nlm.nih.gov/geo/) under accession number GSE141731. Analysis of RNA-sequencing data was performed using the publicly available statistical software package “R.” To remove genes that were lowly expressed in our analysis, we considered only genes that had a relative expression higher than 1 FPKM in both replicates in at least one sample. To identify differentially expressed genes, we considered an expression fold change > 1.5 between treatments for both replicates. Enrichment of biological processes was performed using the Gene Functional Annotation tool from DAVID bioinformatics, Gene Ontology annotation, and Ingenuity Pathway Analysis.

### Confocal microscopy

Cells were grown on gelatin-coated glass coverslips until confluence and incubated with vesicles for the indicated periods. Cells were washed and fixed using 4% (w/v) paraformaldehyde in PBS for 20 min at room temperature. For CD31 expression experiments, cells were permeabilized using 0.25% Triton X-100 in PBS for 5 min, washed twice using 0.1 M glycine in PBS, blocked using 5% goat serum/3% BSA in PBS for 30 min at room temperature and incubated overnight using 1:200 CD31 antibody (Cell Signaling Technology, Danvers, MA, USA) in blocking solution at 4 °C in a humidified chamber. Fixed cells were subsequently washed with PBS, incubated using 1:800 anti-mouse AlexaFluor-594 (Molecular Probes) in blocking solution for 1 h at room temperature then sealed using Prolong Gold DAPI. Confocal images were acquired on a Leica TCS-SP5 inverted microscope using an HCX PL APO 63x/1.4 Oil objective. Excitation system was performed using a 405 diode laser for DAPI, and a 561 nm diode laser for AlexaFluor-594 coupled antibodies using a sequential acquisition at 400 Hz scan speed. Detection bandwidth for CD31 expression experiments was 408–451 nm for DAPI and 586–703 nm for AlexaFluor-594. Images were acquired with the Las-AF software. Final images are 12 bits, 2048 × 2048 with a zoom factor 2 and scale is specified in the figure’s legends. Images were analyzed using FIJI software (NIH). The corrected total cell fluorescence (CTCF) represents the integrated density minus the area of selected cell multiplied by the mean fluorescence of background readings^78^.

### Apoptosis assay by fluorescence microscopy

The following apoptosis assay originated from Dieudé et al.^13^. Fluorescence microscopy of unfixed and unpermeabilized adherent cells stained with Hoechst 33342 (2′-(4-ethoxyphenyl)-5-(4-methyl-1-piperazinyl)-2.5′-bi-1H-benzimidazole (HO), Sigma) and propidium iodide (PI, Invitrogen) was used as described previously^13^. Briefly, cells were grown to confluence in 6-well plates and underwent their respective treatment. HO (1 µg/mL) was added for 10 min at 37 °C then PI was added to a final concentration of 5 µg/mL immediately before analysis (excitation filter, λ = 360–425 nm). Normal, apoptotic and necrotic adherent cell proportions were assessed by an investigator blinded to the experimental conditions in four random fields per condition. Apoptotic cells are characterized by an increase in HO fluorescence indicative of chromatin condensation and an absence of PI positivity indicative of normal cell membrane permeability. Secondary and primary necrotic cells disclose PI positivity demonstrating loss of normal cell membrane permeability.

### Cell cycle assay by flow cytometry

Subconfluent cells were incubated for 12 h according to their respective treatments. Then they were trypsinized, fixed using cold 70% ethanol in PBS, washed in PBS and stained using 20 µg/µL PI—100 µg/mL RNAse A6 in PBS for 20 min at room temperature. The analysis was done by flow cytometry on a FACS-LSRII instrument integrated with FACSDiva software (BD Biosciences).

### Wound healing assay

Cells were cultured on 12-well plates until confluence. The cells were mechanically injured using a P20/P200 pipette tip (three wounds per condition) according to a well-established technique^79^. Marks on the wells enabled us to capture the same fields for each wound (four fields per wound) at various times (at time 0 and 12 h after injury). The wound closure was quantified with TScratch software^80^ from the wound area measured after 12 h compared with the initial wound area, for each wound.

### Endothelial cell tube formation assay

Confluent cells seeded onto 6-W plates were treated for 4 h according to their experimental condition. Subsequently, these cells were detached and re-seeded at a cell density of 130,000 cells/well onto 24-W plates that had been precoated with growth factor reduced Matrigel Matrix (Corning, Corning, NY, USA) and cultured at 37 °C for 7 h under their respective treatment. Each experiment was performed in duplicate for each condition. Capillary-like tubes were captured (four fields per well) and quantified using the Angiogenesis analyzer for ImageJ^81^.

### Endothelial markers characterization

Confluent cells seeded onto T25 flask were treated for 24 h according to their treatment. Cells were stained in suspension with antibodies against endothelial markers, anti-CD31 AlexaFluor 647 (BD Biosciences) and anti-vWF AlexaFluor 405 (ThermoFisher), according to their respective manufacturer’s instructions. The cells were analyzed by flow cytometry on a FACS-LSRII instrument integrated with FACSDiva software (BD Biosciences). Graphs show median fluorescent values (50,000 events/sample).

### siRNA transfection

Cells were plated onto 6-well plates at 2,500 cells per cm^2^. After 72 h, cells were transfected with ON-TARGETplus non-targeting siRNA #1 or 3—SMARTpool, ON-TARGETplus Human RELA siRNA (90 nM)—SMARTpool (Dharmacon, Lafayette, CO, USA) using Magnet Assisted Transfection (MATra) (IBA Lifesciences, Göttingen, Germany) according to manufacturer’s instructions. Briefly, MATra-si Reagent was added to siRNA in a ratio of 1µL:1 µg in Opti-MEM medium (Gibco) and incubated for 25 min at room temperature. The siRNA-beads mixture was added to the supernatant (Opti-MEM) of each well. Then the plate was placed on a magnet plate for 15 min followed by a medium change after 30 min. After 48 h, the cells were processed according to the respective assay.

### Cell lysis, protein isolation and immunoblotting

The following protocols are adapted from Migneault et al.^82^. Total protein extracts were obtained by washing cells twice in ice-cold PBS and lysed for 15 min under agitation at 4 °C in lysis buffer (1% Triton X-100, 150 mM NaCl, 5 mM EDTA, and 50 mM Tris, pH 7.5) supplemented with protease inhibitors (Calbiochem, San Diego, CA, USA) and phosphatase inhibitor cocktails (Sigma). The cells were subsequently scraped with a rubber policeman, collected, and centrifuged at 12,000×*g* for 10 min. Cytoplasmic and nucleic proteins were obtained using the NE-PER Nuclear and Cytoplasmic Extraction Kit according to the manufacturer's instructions (ThermoFisher). Protein concentration was evaluated using the BCA protein assay (ThermoFisher). Proteins were solubilized in sample buffer (25 mM Tris·HCl, pH 6.8, 1% SDS, 0.1% bromophenol blue, 10% glycerol, and 2% β-mercaptoethanol), subjected to SDS–polyacrylamide gel electrophoresis, and transferred electrophoretically onto nitrocellulose membranes. The membranes were blocked with 5% dried fat-free milk in Tris-buffered saline pH 7.4 with 0.05% Tween 20 (TBST) for 1 h at room temperature and then incubated overnight at 4 °C with primary antibody anti-NF-κB (Cell Signaling Technology), anti-p536-NF-κB (Cell Signaling Technology), anti-TBP (Cell Signaling Technology), anti-α-tubulin (Calbiochem) or anti-β-actin (Sigma) in TBST plus 5% milk or 5% BSA. After being washed with TBST, the membranes were incubated with goat anti-rabbit or goat anti-mouse IgG linked to horseradish peroxidase (GE Healthcare, Chicago, IL, USA) for 1 h. After TBST washes, the membranes were incubated with Lumi-Light Western Blotting Substrate (Roche, Basel, Switzerland) or ECL Prime Western Blotting Detection Reagent (GE Healthcare) for 5 min before the luminescent signal was recorded. The intensity of each specific band was quantified with AlphaEaseFC (Alpha Innotech).

### NF-κB luciferase reporter assay

Cells were plated onto 6-well plates at 2,500 cells per cm^2^. After 72 h, cells were transfected with the pGL4.32[luc2P/NF-κB-RE/Hygro] vector (Promega, Madison, WI, USA) containing a NF-κB response element using Magnet Assisted Transfection (MATra) according to the manufacturer’s instructions. pRL-SV40 (Promega), a plasmid expressing *Renilla reniformis* luciferase (RL), was cotransfected for normalization of the Luc response. Briefly, MATra-A Reagent was added to the DNA plasmid in a ratio of 1 µL:3.8 µg pGL4:0.95 µg pRL-SV40 in Opti-MEM medium and incubated for 20 min at room temperature. The DNA-beads mixture was added to the supernatant (Opti-MEM) of each well. Then the plate was placed on a magnet plate for 15 min followed by a medium change after 30 min. After 48 h, the cells were treated for 4 h according to their treatment. Firefly and RL assays were undertaken with the Dual-Luciferase Reporter Assay System, as specified by the manufacturer (Promega). Luminometry measurements were undertaken in a Victor 3 V 1420 Multilabel Counter 1420-040 Microplate Reader (Perkin Elmer, Waltham, MA, USA).

### Statistical analysis

All data are presented as means ± SEM from at least three independent experiments unless otherwise indicated. Data were compared using Student *t *test or stated otherwise in the legend with GraphPad Prism 5 software (GraphPad Software Inc., San Diego, CA, USA). *P* < 0.05 probability was considered to be significant. (*P* values are reported for each experiment.).

## Supplementary information

Supplementary Information
