# Supplementary material for: Apoptotic exosome-like vesicles regulate endothelial gene expression, inflammatory signaling, and function through the NF-κB signaling pathway
Source: Sci Rep. 2020 Jul 28;10:12562. doi: 10.1038/s41598-020-69548-0 (PMC7387353; doi:10.1038/s41598-020-69548-0)
Supplement: Supplementary file 1 — Supplementary Information [file 41598_2020_69548_MOESM1_ESM.pdf]

Supplementary Materials for

Apoptotic exosome-like vesicles regulate endothelial gene expression, inflammatory signaling, and function through the NF-κB signaling pathway.

Francis Migneault[1,2,4], Mélanie Dieudé[1,4], Julie Turgeon[1,4], Déborah Beillevaire[1,2,4], Marie-Pierre Hardy[3,4], Alexandre Brodeur[1], Nicolas Thibodeau[1], Claude Perreault[2,3,4], Marie-Josée Hébert[1,2,4]

1 Research Centre, Centre hospitalier de l'Université de Montréal (CRCHUM).

2 Université de Montréal

3 Institute for Research in Immunology and Cancer (IRIC).

4 Canadian Donation and Transplantation Research Program

* Corresponding author:

Dr Marie-Josée Hébert, CRCHUM,

Tour Viger - R12.414, 900 rue St-Denis, H2X 0A9, Montréal (QC)

Tel:(514) 890-8000 Extension 25393, Fax: (514) 412-7661.

E-mail: marie-josee.hebert@umontreal.ca

Supplementary Materials and Methods

Flow cytometric analyses of EVs. The following flow cytometric analyses originated from Dieudé *et al.* (13). Briefly, fluorescence was used as a trigger signal, and positive fluorescent events were plotted on an SSC/FSC-PMT graph. The ApoExo gate of detection was determined based on the acquisition of sky blue and yellow-green microspheres of sizes 90, 450, 840, 1000, and 3200 nm, and 1000 microspheres were acquired. Conditioned media were labeled for 30 min with V450 probe–conjugated annexin V (diluted 1:50; BD Biosciences) at room temperature in the dark. To process the data quantitatively, a known number of polystyrene microspheres (15-mm diameter; Polysciences) were added to each tube.

Electron microscopy. The following electron microscopy procedures come from Dieudé *et al.* (13). The fraction enriched in apoptotic bodies was fixed in 2% glutaraldehyde-0.1M sodium cacodylate, post-fixed in 1% OsO4, dehydrated in alcohol, processed for flat embedding in Epon 812 and observed with a Zeiss CEM 902 electron microscope. Preparations of ApoExo were fixed in 4% paraformaldehyde in PBS buffer and were contrasted and embedded for whole-mount EM observation. Fixed ApoExo (5 uL) were deposit on a Formvar/carbon-coated grid for 20 min and washed on drops of PBS at RT. The grid was then directly processed for negative staining by transferring it onto a drop of 1% glutaraldehyde for 5 min and washed on several drops of water. Contrasting was performed by transferring the grid onto a drop of 2% uranyl acetate-0.075 M oxalate pH7 solution for 5 min. Finally the grid was transferred onto a drop of 2% methyl cellulose-4 % uranyl acetate (9:1) solution for 10 min on ice and excess solution was blotted with Whatman filter paper and air-dried. Examination was performed with a Philips CM100 electron microscope.

DNA extraction. Isolated extracellular vesicles were resuspended in TRIzol Reagent and total RNA was extracted according to manufacturer instructions. Then, back extraction buffer (BEB) (4 M guanidine thiocyanate, 50 mM sodium citrate, 1 M Tris base) was added to the organic phase (500 µL BEB per 1 mL of initial TRIzol Reagent) and the tube was gently mixed for 10 min following by centrifugation at 13 000 x g for 15 min at 4°C. The aqueous phase was collected and DNA was precipitated by adding isopropanol (400 µL isopropanol per 1 mL of initial TRIzol Reagent) and washed once with 70% ethanol. The pellet was resuspended in deionized water. dsDNA was quantified using the Qubit dsDNA HS Assay Kit (ThermoFisher) according to manufacturer instructions.

Table S1. List of the differentially expressed genes between endothelial cells exposed to ApoExo compared to vehicle-treated cells

Figure S1. Serum starvation increases caspase-3 activation and chromatin condensation in endothelial cells. (**A**) Evaluation by Hoescht and Propidium iodide (HO/PI) staining of apoptotic or necrotic cells in endothelial cells exposed for 4 h to normal media or serum starvation. HO/PI experiments expressed as the percentage of apoptosis ± SEM. n = 9 for each condition. P value obtained by unpaired t-test. (**B**) Endothelial cells were exposed to normal media or serum starvation for 4 h and cleavage of caspase-3 was assessed. Cleaved caspase-3 was quantified by the cleaved caspase-3-to-β-actin ratio and expressed as arbitrary units ± SEM. Representative immunoblot of the cleavage of caspase 3 after 4 h of serum starvation is presented. n = 3 for each condition. P value was obtained by unpaired t-test.

Figure S2. Characterization of the apoptotic exosome-like exosome (ApoExo). (**A**) Workflow for generation and isolation of ApoExo. (**B**) Flow cytometric quantifications of annexin V+ apoptotic bodies and ApoExo detected in the supernatant of conditioned endothelial cells ± SEM. n = 12 for each condition. (**C**) Total RNA quantification of apoptotic bodies and ApoExo detected in the supernatant of conditioned endothelial cells expressed as ng/mL of supernatant ± SEM. n = 7 for each condition. (**D**) Total protein quantification of apoptotic bodies and ApoExo detected in the supernatant of conditioned endothelial cells expressed as μg/mL of supernatant ± SEM. n = 20 for each condition. (**E**) Electron micrographs of apoptotic bodies and ApoExo released by serum-starved HUVECs and isolated by sequential ultracentrifugation. (**F**) Immunoblot analysis of different protein markers in ApoExo and apoptotic bodies from supernatant of conditioned endothelial cells. n ≥ 3 for each condition. Representative immunoblots are presented.

Figure S3. Open wound areas are consistent between the experimental conditions. Serum-starved endothelial cells were mechanically injured and exposed to the vehicle (Ctrl) or apoptotic exosome-like vesicles (ApoExo) (Scale bar: 200 µm). Percentage of open wound areas at 0 h performed with TScratch software. Data are shown as % of the open wound area. n ≥ 6 for each condition. P values obtained by one-way ANOVA.

Figure S4. Apoptotic exosome-like vesicles decrease several angiogenic parameters. Serum-starved endothelial cells were exposed to the vehicle (Ctrl) or apoptotic exosome-like vesicles (ApoExo) and capillary-like structures were quantified following a 7 h treatment on extracellular matrix. Capillaries density is depicted as the number of nodes, junctions and segment length per field ± SEM. n ≥ 3 for each condition. P values obtained by unpaired t-test.

Figure S5. Modulation of endothelial functions and NF-κB activation are specific to apoptotic exosome-like vesicles (ApoExo). (**A**) Serum-starved endothelial cells were mechanically injured and exposed to the vehicle (Ctrl), ApoExo, apoptotic bodies, exosomes (ExoN) isolated from the 200 000 x g centrifugation of conditioned media of non-apoptotic endothelial cells, ApoExo-depleted supernatant post-200 000 x g centrifugation or ApoExo treated with 0.05% Triton X-100. Wound closure was followed over a 12 h period. Wound healing assay expressed as the percentage of wound closure ± SEM. n = 3 for each condition. P values were obtained by one-way ANOVA and Bonferroni's *post hoc* test. (**B**) Serum-starved endothelial cells were exposed to the vehicle (Ctrl), ApoExo, apoptotic bodies or exosomes (ExoN) isolated from the 200 000 x g centrifugation of conditioned media of non-apoptotic endothelial cells and capillary-like structures were quantified following a 7 h treatment on extracellular matrix. Capillaries density is depicted as the number of segment per field ± SEM. n = 3 for each condition. P values obtained by one-way ANOVA and Bonferroni's *post hoc* test. (**C**) Serum-starved endothelial cells were exposed to the

vehicle (Ctrl), ApoExo, apoptotic bodies, exosomes (ExoN) isolated from the 200 000 x g centrifugation of conditioned media of non-apoptotic endothelial cells, ApoExo-depleted supernatant post-200 000 x g centrifugation or ApoExo treated with 0.05% Triton X-100 for 4 h and phosphorylation of NF-κB at Ser536 (p536-NF-κB) was assessed. NF-κB phosphorylation was quantified by the phospho-to-total ratio and expressed as arbitrary units ± SEM. n = 3 for each condition. Representative immunoblots cropped from the same gel are presented. P values were obtained by one-way ANOVA and Bonferroni's *post hoc* test.

Figure S6. Apoptotic exosome-like vesicles do not modulate the expression of mesenchymal markers in endothelial cells. Apoptotic exosome-like vesicles do not induce the expression of α-SMA or S100A4 in endothelial cells. α-SMA and S100A4 expression by flow cytometry analysis of serum-starved endothelial cells exposed for 24 h to the vehicle (Ctrl) or apoptotic exosome-like vesicles (ApoExo). Flow cytometry experiments expressed as the percentage of vehicle-treated cells median fluorescence intensity (50,000 events/sample) ± SEM (right). Representative gates of α-SMA and S100A4 expression are depicted (left). n = 3 for each condition.

Figure S7. NF-κB inhibition impaired pro-migratory phenotype induced by apoptotic exosome-like vesicles. Serum-starved endothelial cells treated with vehicle or Celastrol (NF-κB inh.) were mechanically injured and exposed to the vehicle (Ctrl) or apoptotic exosome-like vesicles (ApoExo). Wound closure was followed over a 12 h period (Scale bar: 200 μm). Wound healing assay expressed as the percentage of wound closure ± SEM. n = 3 for each condition. Representative pictures and immunoblot of the inhibition of phosphorylation of NF-κB at Ser536 (pNF-κB) at 12 h post-injury are presented. P values were obtained by one-way ANOVA and Bonferroni's *post hoc* test.

Figure S8. NF-κB inhibition restores angiogenic parameters decreased by apoptotic exosome-like vesicles in endothelial cells. NF-κB knock-down restore tubules formation decreased by apoptotic exosome-like vesicles. Serum-starved endothelial cells transfected with Ctrl or NF-κB siRNA 90 nM were exposed to the vehicle (Ctrl) or apoptotic exosome-like vesicles (ApoExo) and capillary-like structures were quantified following a 7 h treatment on extracellular matrix. Capillaries density is depicted as the number of nodes, junctions and segment length per field ± SEM. n = 4 for each condition. P values were obtained by one-way ANOVA and Bonferroni's *post hoc* test.

Figure S9. Validation of RNA-seq data using RT-qPCR. The expression of ICAM-1, VCAM-1, and E-Selectin using RNA-sequencing data and RT-qPCR analysis on serum-starved endothelial cells exposed to ApoExo was assessed. ApoExo did not modulate the expression of surface adhesion molecules compared to control both by RNA-sequencing analysis and RT-qPCR. n = 2 for each condition.

Figure S10. Modulation of endothelial phenotype by apoptotic exosome-like vesicles (ApoExo) is proteasome-independent. Serum-starved endothelial cells were mechanically injured and exposed to the vehicle (Ctrl) or ApoExo from endothelial cells treated with the proteasome inhibitor bortezomib or vehicle. Wound closure was followed over a 12 h period. Wound healing assay expressed as the percentage of wound closure ± SEM. n = 3 for each condition. P values were obtained by one-way ANOVA and Bonferroni's *post hoc* test. CD31 expression by flow cytometry analysis of serum-starved endothelial cells exposed for 24 h to the vehicle (Ctrl) or ApoExo from endothelial cells treated with the proteasome inhibitor bortezomib or vehicle. Flow cytometry experiments expressed as the percentage of vehicle-treated cells

median fluorescence intensity (50,000 events/sample) ± SEM (right). n = 3 for each condition. P values were obtained by one-way ANOVA and Bonferroni's *post hoc* test.

Figure S11. Apoptotic exosome-like vesicles (ApoExo) are not enriched in double-strand DNA. Double-strand DNA quantification of apoptotic bodies and ApoExo detected in the supernatant of conditioned endothelial cells expressed as pg/mL of supernatant ± SEM. n = 2 for each condition.

Figure S12. Source data, unedited gels from Figure 4.

Figure S13. Source data, unedited gels from Figure 5.

Figure S14. Source data, unedited gels from Figure S1.

Figure S15. Source data, unedited gels from Figure S2.

Figure S16. Source data, unedited gels from Figure S5.

Table S1

Gene.ID	SS.2	SS	Nano.2	Nano	Gene	log2FC	log2FC.2	log2FC mean	Gene type
		FPKM							
ENSG00000224043	1,19	2,70	0,72	1,47	CCNT2-AS1	-0,88	-0,72	-0,80	antisense
ENSG00000224063	0,72	0,18	1,35	1,65	AC007319.1	3,19	0,91	2,05	antisense
ENSG00000227751	0,31	0,71	1,61	3,00	RP1-20B21.4	2,08	2,37	2,23	antisense
ENSG00000254911	2,35	0,58	4,56	1,75	SCARNA9	1,59	0,96	1,27	antisense
ENSG00000262533	0,37	0,44	1,15	1,67	RP11-667K14.4	1,92	1,63	1,78	antisense
ENSG00000266341	0,88	0,00	2,22	2,24	RP5-890E16.4	11,13	1,33	6,23	antisense
ENSG00000175772	0,70	0,86	1,07	1,53	LINC01106	0,83	0,61	0,72	lincRNA
ENSG00000205663	0,85	0,64	1,39	1,51	RP11-706O15.5	1,24	0,71	0,97	lincRNA
ENSG00000226007	0,30	0,41	1,17	1,10	RP11-211N8.2	1,42	1,96	1,69	lincRNA
ENSG00000236914	1,58	1,54	0,93	0,71	RP11-1008C21.2	-1,12	-0,76	-0,94	lincRNA
ENSG00000258610	2,26	1,16	0,00	0,00	RP11-488C13.7	-10,18	-11,14	-10,66	lincRNA
ENSG00000260923	3,24	4,07	1,46	2,59	AC137934.1	-0,65	-1,15	-0,90	lincRNA
ENSG00000263394	2,00	1,41	1,15	0,90	RP11-160E2.19	-0,65	-0,80	-0,72	lincRNA
ENSG00000270103	4,46	2,59	9,94	4,41	RNU11	0,77	1,16	0,96	lincRNA
ENSG00000271737	1,04	1,34	0,50	0,73	CTB-113I20.2	-0,88	-1,06	-0,97	lincRNA
ENSG00000274818	0,91	0,59	1,91	1,29	RP1-292L20.3	1,13	1,07	1,10	lincRNA
ENSG00000275496	7,88	13,16	3,17	7,33	CH507-24F1.2	-0,84	-1,31	-1,08	lincRNA
ENSG00000199133	4,05	5,65	0,00	2,14	MIRLET7D	-1,40	-11,98	-6,69	miRNA
ENSG00000207548	0,00	0,00	4,90	1,74	MIR217	10,77	12,26	11,51	miRNA
ENSG00000264248	1,80	0,00	3,00	1,26	AL603831.1	10,30	0,74	5,52	miRNA
ENSG00000265238	18,79	3,01	0,00	0,00	MIR3614	-11,56	-14,20	-12,88	miRNA
ENSG00000266192	0,00	0,00	5,85	1,96	MIR1260B	10,94	12,51	11,73	miRNA
ENSG00000273917	0,04	0,00	2,74	7,12	AC126544.1	12,80	6,06	9,43	miRNA
ENSG00000275668	2,91	0,00	9,70	1,68	MIR29C	10,72	1,74	6,23	miRNA
ENSG00000276078	10,37	11,48	0,00	3,90	AC091154.1	-1,56	-13,34	-7,45	miRNA
ENSG00000278371	3,66	2,20	2,04	0,96	AL442127.1	-1,20	-0,84	-1,02	miRNA
ENSG00000280605	0,00	0,00	3,71	2,90	AP001107.1	11,50	11,86	11,68	miRNA
ENSG00000280849	0,00	0,00	4,70	1,69	AC005339.1	10,72	12,20	11,46	miRNA
ENSG00000280874	0,56	0,00	2,52	1,82	AC239868.2	10,83	2,17	6,50	miRNA
ENSG00000281134	0,56	0,00	2,52	1,82	AC239868.3	10,83	2,17	6,50	miRNA
ENSG00000200391	2,81	4,28	0,00	0,00	Y_RNA	-12,06	-11,46	-11,76	misc_RNA
ENSG00000273541	1,58	0,55	3,28	2,09	DLEU1_2	1,92	1,05	1,49	misc_RNA
ENSG00000273961	0,00	4,90	6,96	8,99	HOXA11-AS1_1	0,88	12,77	6,82	misc_RNA
ENSG00000274396	18,28	30,34	12,03	15,40	HOTAIRM1_4	-0,98	-0,60	-0,79	misc_RNA

ENSG00000274845	0,00	0,00	1,71	1,53	uc_338	10,58	10,74	10,66	misc_RNA
ENSG00000275134	1,44	0,00	3,11	3,08	MEG8_2	11,59	1,11	6,35	misc_RNA
ENSG00000281789	0,00	0,00	11,72	6,09	Y_RNA	12,57	13,52	13,04	misc_RNA
ENSG00000178715	0,83	2,84	2,52	4,88	RP11-169K16.8	0,78	1,60	1,19	processed_pseudogene
ENSG00000183586	0,86	2,73	2,19	5,31	HMGN3P1	0,96	1,35	1,15	processed_pseudogene
ENSG00000214857	1,43	4,58	0,60	1,54	SHFM1P1	-1,57	-1,25	-1,41	processed_pseudogene
ENSG00000223723	0,73	0,72	1,15	1,37	BX842568.2	0,93	0,65	0,79	processed_pseudogene
ENSG00000224365	16,65	9,02	28,31	19,27	RP11-252P18.1	1,10	0,77	0,93	processed_pseudogene
ENSG00000225486	0,99	0,86	1,73	2,02	RP11-301G21.1	1,23	0,80	1,02	processed_pseudogene
ENSG00000228399	2,08	2,10	3,63	8,87	RP4-575N6.2	2,08	0,80	1,44	processed_pseudogene
ENSG00000229023	0,65	0,78	1,36	2,33	AC067945.3	1,58	1,06	1,32	processed_pseudogene
ENSG00000229230	1,64	4,47	0,00	2,25	MT1P3	-0,99	-10,68	-5,84	processed_pseudogene
ENSG00000231702	2,51	1,61	0,00	0,73	RP11-54O7.10	-1,14	-11,29	-6,22	processed_pseudogene
ENSG00000232727	1,55	1,86	2,44	3,88	YWHAEP1	1,06	0,65	0,86	processed_pseudogene
ENSG00000233205	1,06	0,71	1,84	1,11	AC108479.2	0,64	0,80	0,72	processed_pseudogene
ENSG00000235028	1,19	2,35	1,82	4,29	HMGN1P30	0,87	0,61	0,74	processed_pseudogene
ENSG00000235043	5,06	4,04	8,11	6,07	TECRP1	0,59	0,68	0,63	processed_pseudogene
ENSG00000235211	0,00	0,00	4,97	1,10	TMSB10P2	10,10	12,28	11,19	processed_pseudogene
ENSG00000235378	0,00	11,75	14,97	21,26	MRPS10P1	0,86	13,87	7,36	processed_pseudogene
ENSG00000235579	0,41	0,00	1,39	1,27	AC007283.4	10,31	1,76	6,04	processed_pseudogene
ENSG00000236281	2,17	1,86	1,32	1,18	AC093106.5	-0,66	-0,72	-0,69	processed_pseudogene
ENSG00000236312	1,87	4,13	3,71	6,70	RPL34P34	0,70	0,99	0,84	processed_pseudogene
ENSG00000237701	0,29	1,00	1,26	1,95	ATP5JP1	0,96	2,12	1,54	processed_pseudogene
ENSG00000240616	1,20	5,02	2,13	8,39	RPS6P25	0,74	0,83	0,78	processed_pseudogene
ENSG00000241464	1,64	3,27	0,00	1,32	RPL39P38	-1,31	-10,68	-5,99	processed_pseudogene
ENSG00000242634	0,52	1,22	1,47	3,18	RPS24P16	1,38	1,50	1,44	processed_pseudogene
ENSG00000248794	0,61	0,51	1,15	1,22	CTD-2248H3.1	1,26	0,91	1,09	processed_pseudogene
ENSG00000249986	0,80	2,21	1,67	4,09	YWHAQP6	0,89	1,06	0,97	processed_pseudogene
ENSG00000250202	0,93	1,19	1,60	2,05	RP11-397E7.2	0,78	0,78	0,78	processed_pseudogene
ENSG00000256148	0,00	0,00	1,17	1,92	RP11-809N8.5	10,91	10,19	10,55	processed_pseudogene
ENSG00000261588	0,00	1,97	1,51	3,81	AC002310.17	0,95	10,56	5,76	processed_pseudogene
ENSG00000267340	0,00	0,00	1,37	1,27	RP11-242D8.3	10,31	10,42	10,37	processed_pseudogene
ENSG00000223745	0,87	1,31	2,13	2,07	RP4-717I23.3	0,66	1,29	0,98	processed_transcript
ENSG00000259244	1,32	0,82	2,77	1,90	RP11-182J1.12	1,21	1,07	1,14	processed_transcript
ENSG00000273184	2,50	2,25	5,50	3,88	RP11-212P7.3	0,79	1,14	0,96	processed_transcript
ENSG00000073331	1,57	0,74	3,07	1,32	ALPK1	0,83	0,97	0,90	protein_coding
ENSG00000101695	0,90	0,49	1,47	1,35	RNF125	1,46	0,71	1,08	protein_coding

ENSG00000107821	0,62	0,50	1,01	1,05	KAZALD1	1,07	0,70	0,89	protein_coding
ENSG00000110057	3,85	4,40	2,42	2,62	UNC93B1	-0,75	-0,67	-0,71	protein_coding
ENSG00000162878	1,08	0,66	1,69	1,11	PKDCC	0,75	0,65	0,70	protein_coding
ENSG00000168685	1,11	2,57	0,47	1,58	IL7R	-0,70	-1,24	-0,97	protein_coding
ENSG00000172058	9,77	5,51	5,05	3,08	SERF1A	-0,84	-0,95	-0,90	protein_coding
ENSG00000184719	3,00	2,53	1,97	1,45	RNLS	-0,80	-0,61	-0,70	protein_coding
ENSG00000185019	1,47	1,46	2,22	2,33	UBOX5	0,67	0,59	0,63	protein_coding
ENSG00000185513	0,81	0,67	1,39	1,03	L3MBTL1	0,62	0,78	0,70	protein_coding
ENSG00000196754	1,99	3,29	1,32	2,12	S100A2	-0,63	-0,59	-0,61	protein_coding
ENSG00000197779	1,08	0,95	1,75	1,50	ZNF81	0,66	0,70	0,68	protein_coding
ENSG00000198064	0,71	0,50	1,63	1,12	RP11-347C12.1	1,16	1,20	1,18	protein_coding
ENSG00000235194	1,88	1,22	2,87	2,37	PPP1R3E	0,96	0,61	0,78	protein_coding
ENSG00000240891	3,14	2,28	1,97	1,41	PLCXD2	-0,69	-0,67	-0,68	protein_coding
ENSG00000243414	1,97	1,18	0,99	0,68	TICAM2	-0,79	-0,99	-0,89	protein_coding
ENSG00000256977	8,04	2,08	4,80	0,00	LIMS3	-11,02	-0,74	-5,88	protein_coding
ENSG00000259316	12,82	1,75	20,99	3,93	CTD-2116N17.1	1,17	0,71	0,94	protein_coding
ENSG00000259332	1,71	3,49	0,43	1,65	ST20-MTHFS	-1,08	-1,99	-1,53	protein_coding
ENSG00000260537	3,68	1,41	2,06	0,00	RP11-529K1.3	-10,46	-0,84	-5,65	protein_coding
ENSG00000267740	2,09	1,51	1,39	0,66	AC024592.12	-1,19	-0,59	-0,89	protein_coding
ENSG00000267952	3,49	6,91	7,63	11,13	CTD-2207O23.12	0,69	1,13	0,91	protein_coding
ENSG00000270149	1,35	1,42	2,40	4,47	RP11-544M22.13	1,65	0,83	1,24	protein_coding
ENSG00000272196	1,67	0,00	2,70	5,51	HIST2H2AA4	12,43	0,69	6,56	protein_coding
ENSG00000279274	0,58	0,79	1,18	2,06	AC012005.3	1,38	1,02	1,20	protein_coding
ENSG00000279473	0,66	1,25	1,17	2,85	AC009065.4	1,19	0,83	1,01	protein_coding
ENSG00000280755	0,00	0,23	1,44	1,58	AC009950.1	2,77	10,49	6,63	protein_coding
ENSG00000249784	23,37	18,08	6,00	10,05	SCARNA22	-0,85	-1,96	-1,40	scaRNA
ENSG00000252481	0,97	0,44	2,01	1,15	SCARNA13	1,38	1,05	1,22	scaRNA
ENSG00000242590	5,98	5,84	9,06	9,61	RP11-54O7.14	0,72	0,60	0,66	sense_intronic
ENSG00000250135	0,83	1,02	1,51	3,06	RP4-622L5.2	1,58	0,86	1,22	sense_intronic
ENSG00000261783	1,24	1,05	0,59	0,57	RP11-252K23.2	-0,88	-1,07	-0,98	sense_intronic
ENSG00000269925	1,04	0,49	1,65	1,85	RP3-467L1.6	1,91	0,67	1,29	sense_intronic
ENSG00000269970	0,93	0,00	1,58	1,02	RP11-498E2.9	10,00	0,76	5,38	sense_intronic
ENSG00000271452	0,40	0,57	1,00	1,77	RP11-342K6.2	1,63	1,32	1,48	sense_intronic
ENSG00000243960	4,19	7,91	1,55	3,90	RP11-552M11.4	-1,02	-1,43	-1,23	sense_overlapping
ENSG00000250917	0,40	0,76	1,13	1,37	RP4-785G19.5	0,85	1,50	1,17	sense_overlapping
ENSG00000206621	0,00	0,00	14,14	6,87	SNORD116-14	12,75	13,79	13,27	snoRNA
ENSG00000206634	1,36	2,41	0,00	0,00	SNORA22	-11,24	-10,41	-10,82	snoRNA

ENSG00000207063	21,07	8,55	0,00	3,04	SNORD116-1	-1,49	-14,36	-7,93	snoRNA
ENSG00000207067	1,44	0,00	4,82	1,08	SNORA72	10,08	1,74	5,91	snoRNA
ENSG00000207279	8,49	19,72	0,00	6,87	SNORD116-24	-1,52	-13,05	-7,29	snoRNA
ENSG00000207375	0,00	0,00	14,14	3,43	SNORD116-23	11,74	13,79	12,77	snoRNA
ENSG00000207445	3,99	0,00	6,68	2,50	SNORD15B	11,29	0,74	6,02	snoRNA
ENSG00000208892	1,29	2,30	0,00	0,00	SNORA49	-11,17	-10,33	-10,75	snoRNA
ENSG00000212283	29,68	33,15	4,50	18,14	SNORD89	-0,87	-2,72	-1,80	snoRNA
ENSG00000221164	3,26	2,79	13,62	10,58	SNORA11	1,92	2,06	1,99	snoRNA
ENSG00000221491	1,25	2,25	0,00	0,00	SNORA34	-11,14	-10,29	-10,71	snoRNA
ENSG00000222489	1,91	1,79	0,00	0,00	SNORA79	-10,81	-10,90	-10,85	snoRNA
ENSG00000238832	0,00	0,00	1,71	2,54	snoU109	11,31	10,74	11,03	snoRNA
ENSG00000239039	29,78	23,47	7,11	0,00	SNORD13	-14,52	-2,07	-8,29	snoRNA
ENSG00000254341	0,61	0,00	10,03	8,46	SNORD87	13,05	4,04	8,54	snoRNA
ENSG00000272034	0,00	0,00	15,09	3,58	SNORD14A	11,81	13,88	12,84	snoRNA
ENSG00000275146	0,00	0,00	31,01	11,31	snoU2_19	13,47	14,92	14,19	snoRNA
ENSG00000199347	44,93	31,13	28,57	16,96	RNU5E-1	-0,88	-0,65	-0,76	snRNA
ENSG00000199568	17,43	14,60	74,82	25,41	RNU5A-1	0,80	2,10	1,45	snRNA
ENSG00000200156	14,94	4,87	29,10	15,24	RNU5B-1	1,65	0,96	1,30	snRNA
ENSG00000200469	4,00	5,73	0,00	0,00	RNU6-112P	-12,48	-11,97	-12,23	snRNA
ENSG00000252311	0,00	0,00	3,00	1,26	RNU1-103P	10,30	11,55	10,93	snRNA
ENSG00000280320	0,48	1,12	1,68	3,00	RP11-629N8.5	1,42	1,81	1,61	TEC
ENSG00000227682	2,04	4,56	3,11	8,57	ATP5A1P2	0,91	0,61	0,76	transcribed_processed_pseudogene
ENSG00000241434	1,61	1,52	0,00	0,62	CTD-2224J9.4	-1,29	-10,65	-5,97	transcribed_processed_pseudogene
ENSG00000259516	1,05	1,43	1,73	2,41	ANP32AP1	0,75	0,72	0,74	transcribed_processed_pseudogene
ENSG00000274026	2,09	1,58	0,77	0,74	FAM27E3	-1,09	-1,44	-1,27	transcribed_processed_pseudogene
ENSG00000189136	3,43	3,86	2,22	2,22	UBE2Q2P1	-0,80	-0,63	-0,71	transcribed_unprocessed_pseudogene
ENSG00000205918	0,20	0,54	1,31	1,05	PDPK2P	0,96	2,71	1,83	transcribed_unprocessed_pseudogene
ENSG00000228782	2,60	3,09	4,14	4,75	CTD-2026D20.3	0,62	0,67	0,65	transcribed_unprocessed_pseudogene
ENSG00000243406	1,59	1,73	0,88	1,10	MRPS31P5	-0,65	-0,85	-0,75	transcribed_unprocessed_pseudogene
ENSG00000196302	1,38	1,48	0,00	0,53	RP11-497H16.5	-1,48	-10,43	-5,96	unprocessed_pseudogene
ENSG00000248126	2,31	5,94	15,43	14,07	CTD-2012J19.2	1,24	2,74	1,99	unprocessed_pseudogene

		%
protein_coding	27	19
pseudogene	39	28
lincRNA/lncRNA	23	17
antisense	6	4
snRNA	24	17
misc_RNA	7	5
miRNA	13	9
	139	

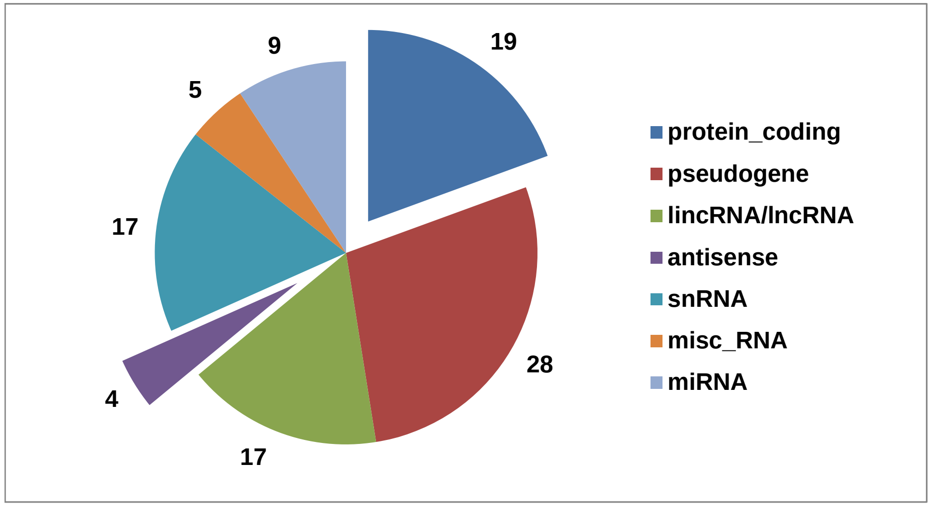

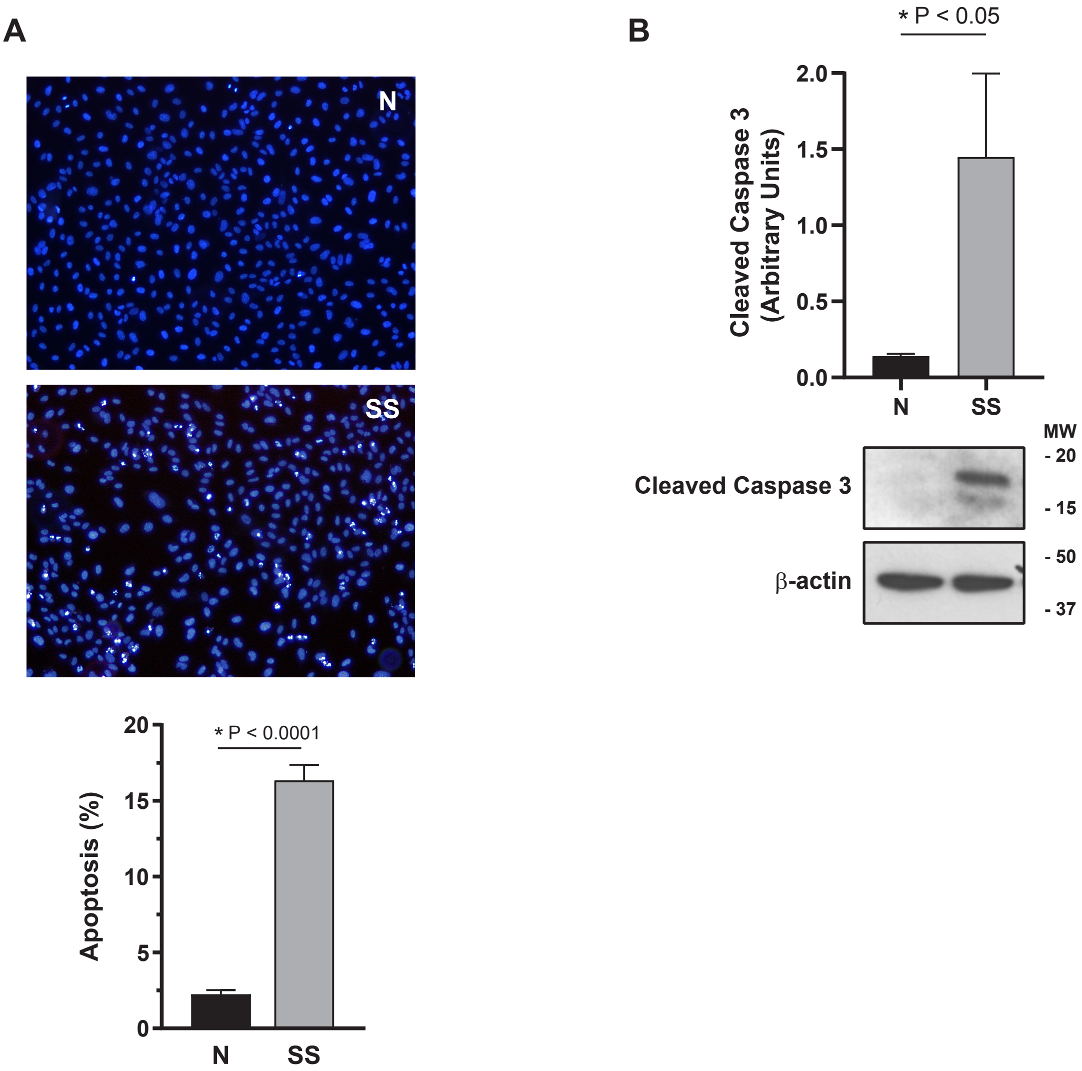
A
N
SS
20
15
10
5
0
Apoptosis (%)
* P < 0.0001
N
SS
B
* P < 0.05
2.0
1.5
1.0
0.5
0.0
Cleaved Caspase 3
(Arbitrary Units)
N
SS
MW
- 20
- 15
- 50
- 37
Cleaved Caspase 3
β-actin

Figure S1

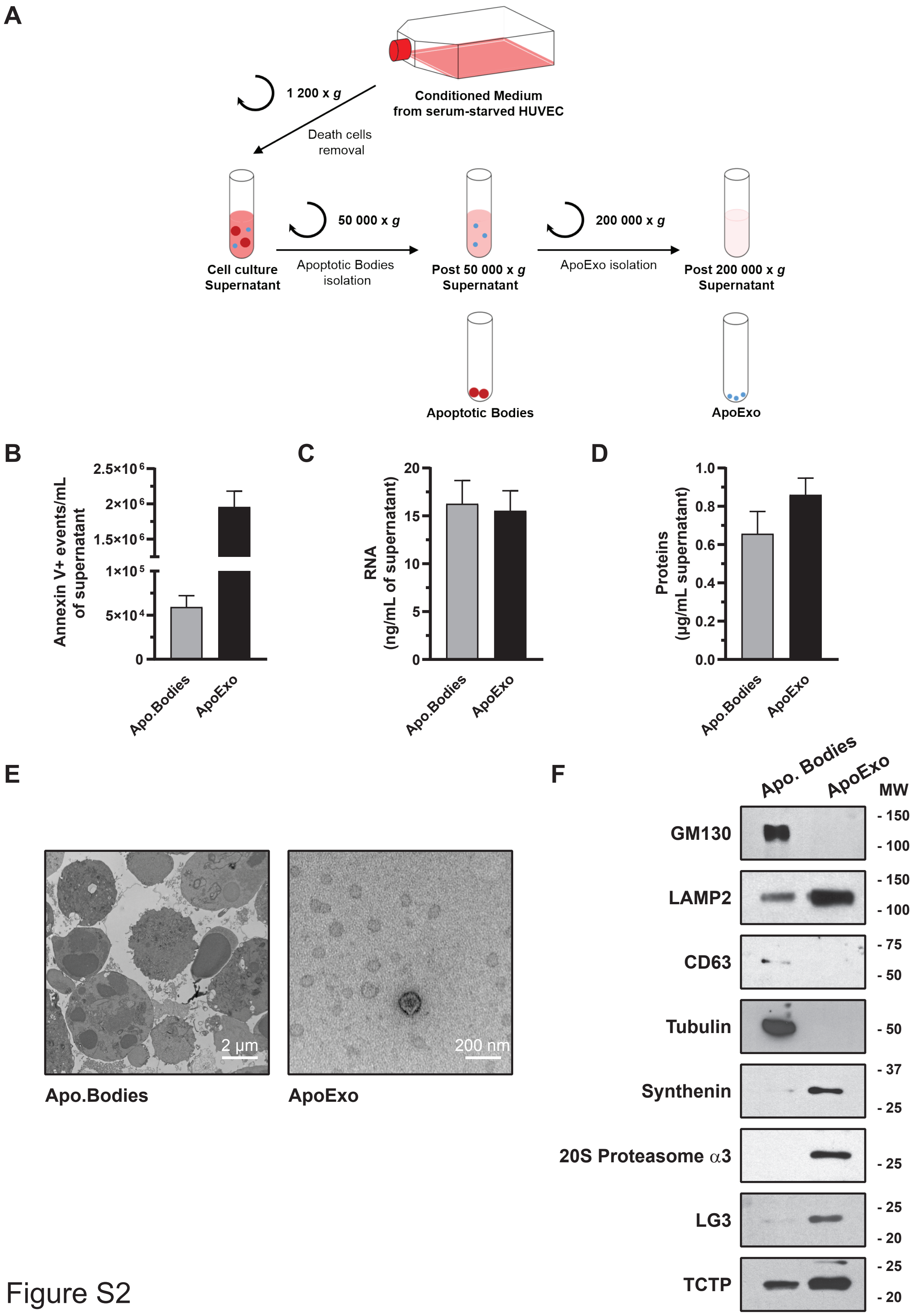
A
1 200 x g
Conditioned Medium
from serum-starved HUVEC
Death cells
removal
50 000 x g
200 000 x g
Cell culture
Supernatant
Apoptotic Bodies
isolation
Post 50 000 x g
Supernatant
ApoExo isolation
Post 200 000 x g
Supernatant
Apoptotic Bodies
ApoExo
B
Annexin V+ events/mL
of supernatant
2.5×10^6
2×10^6
1.5×10^6
1×10^5
5×10^4
0
Apo.Bodies
ApoExo
C
RNA
(ng/mL of supernatant)
20
15
10
5
0
Apo.Bodies
ApoExo
D
Proteins
(µg/mL supernatant)
1.0
0.8
0.6
0.4
0.2
0.0
Apo.Bodies
ApoExo
E
2 µm
200 nm
Apo.Bodies
ApoExo
F
Apo. Bodies
ApoExo
MW
GM130
LAMP2
CD63
Tubulin
Synthenin
20S Proteasome α3
LG3
TCTP
- 150
- 100
- 150
- 100
- 75
- 50
- 50
- 37
- 25
- 25
- 25
- 20
- 25
- 20
Figure S2

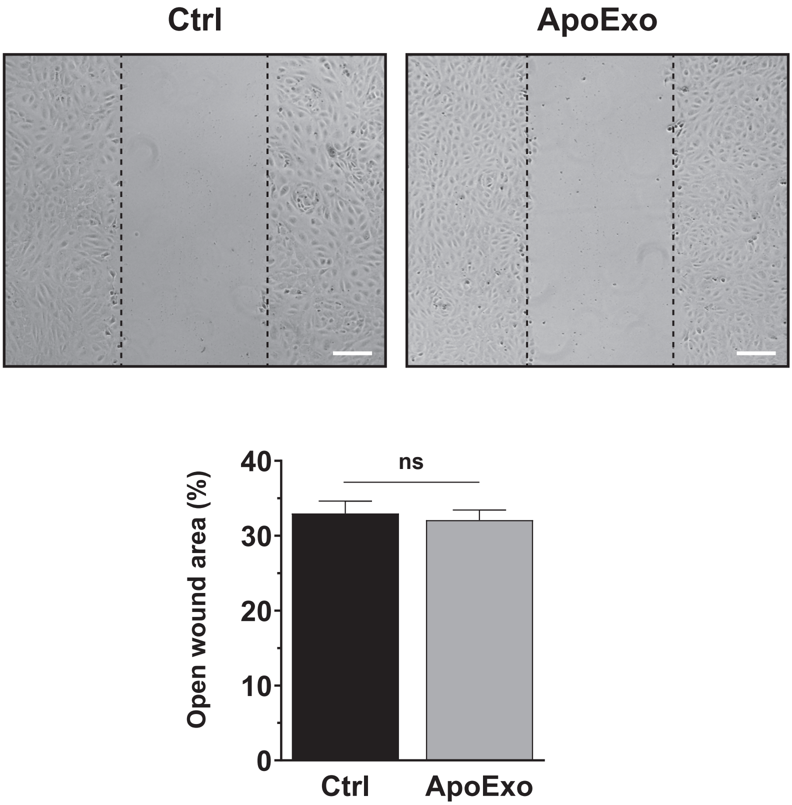

Figure S3

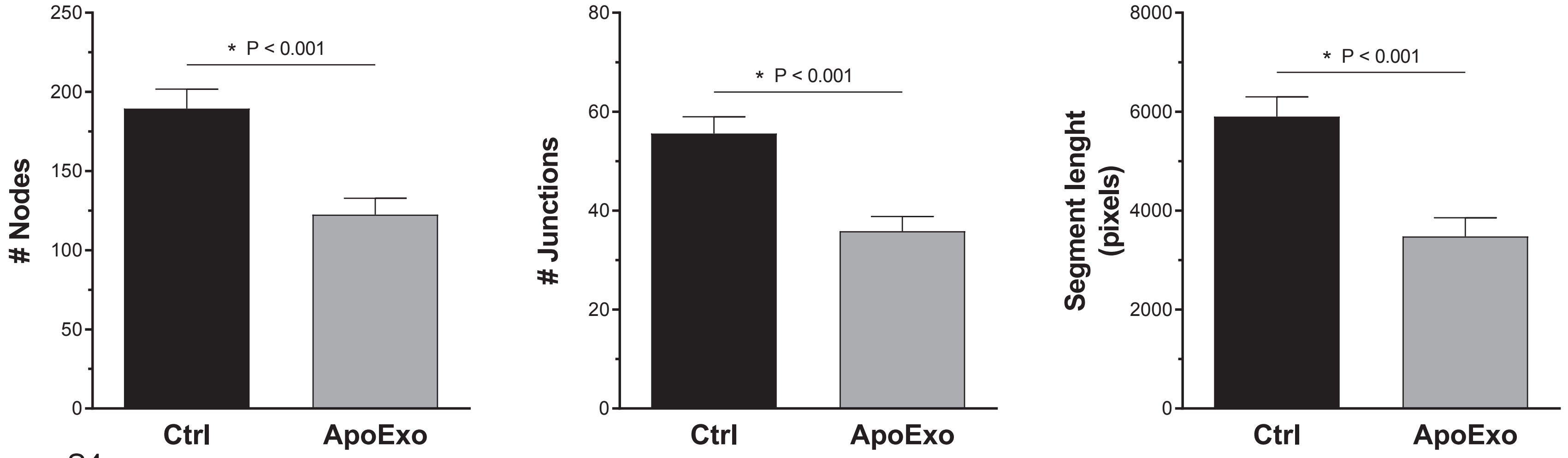
250
200
150
100
50
0
Nodes
* P < 0.001
Ctrl
ApoExo
80
60
40
20
0
Junctions
* P < 0.001
Ctrl
ApoExo
8000
6000
4000
2000
0
Segment lenght (pixels)
* P < 0.001
Ctrl
ApoExo
Figure S4

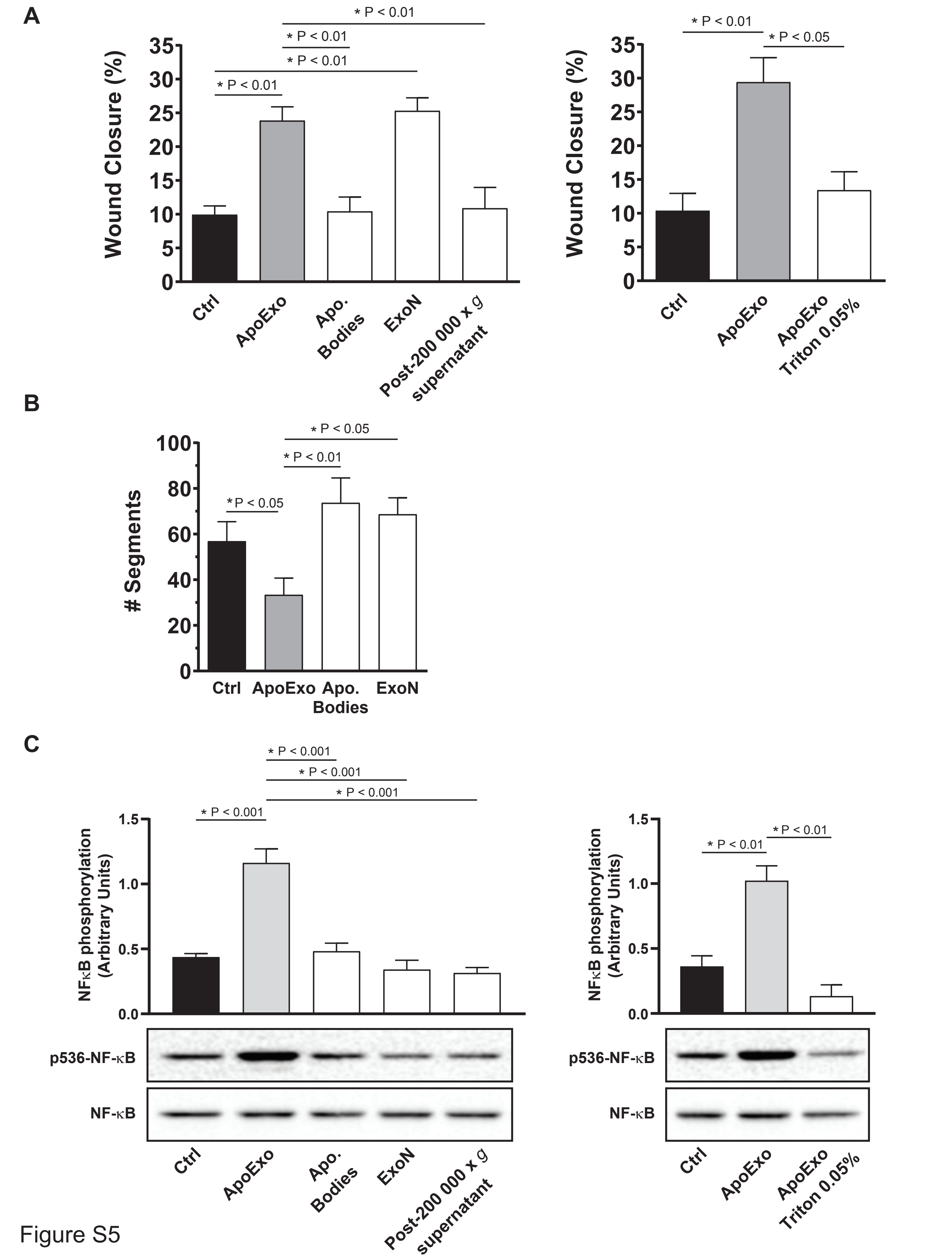

A
Wound Closure (%)
* P < 0.01
* P < 0.01
* P < 0.01
* P < 0.01
Ctrl
ApoExo
Apo. Bodies
ExoN
Post-200 000 x g supernatant
* P < 0.01
* P < 0.05
ApoExo Triton 0.05%
B
Segments
*P < 0.05
* P < 0.01
* P < 0.05
C
NFκB phosphorylation (Arbitrary Units)
* P < 0.001
* P < 0.001
* P < 0.001
* P < 0.001
* P < 0.01
* P < 0.01
p536-NF-κB
NF-κB

Figure S5

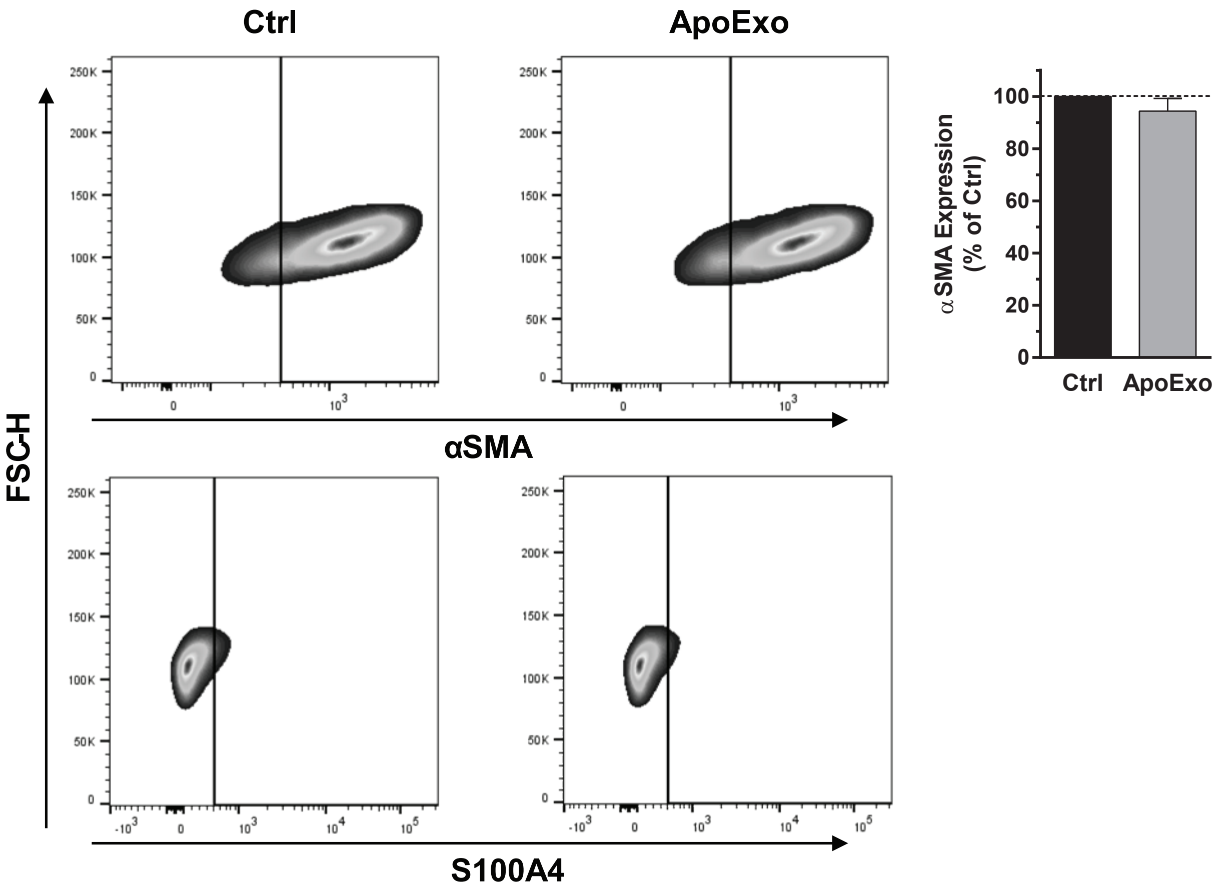

Ctrl
ApoExo
FSC-H
αSMA
S100A4
αSMA Expression (% of Ctrl)
Ctrl
ApoExo

Figure S6

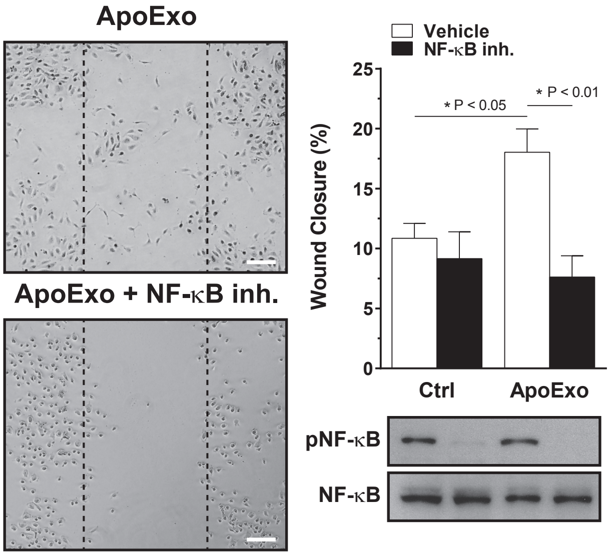
ApoExo
ApoExo + NF-κB inh.
Vehicle
NF-κB inh.
Wound Closure (%)
25
20
15
10
5
0
* P < 0.05
* P < 0.01
Ctrl
ApoExo
pNF-κB
NF-κB

Figure S7

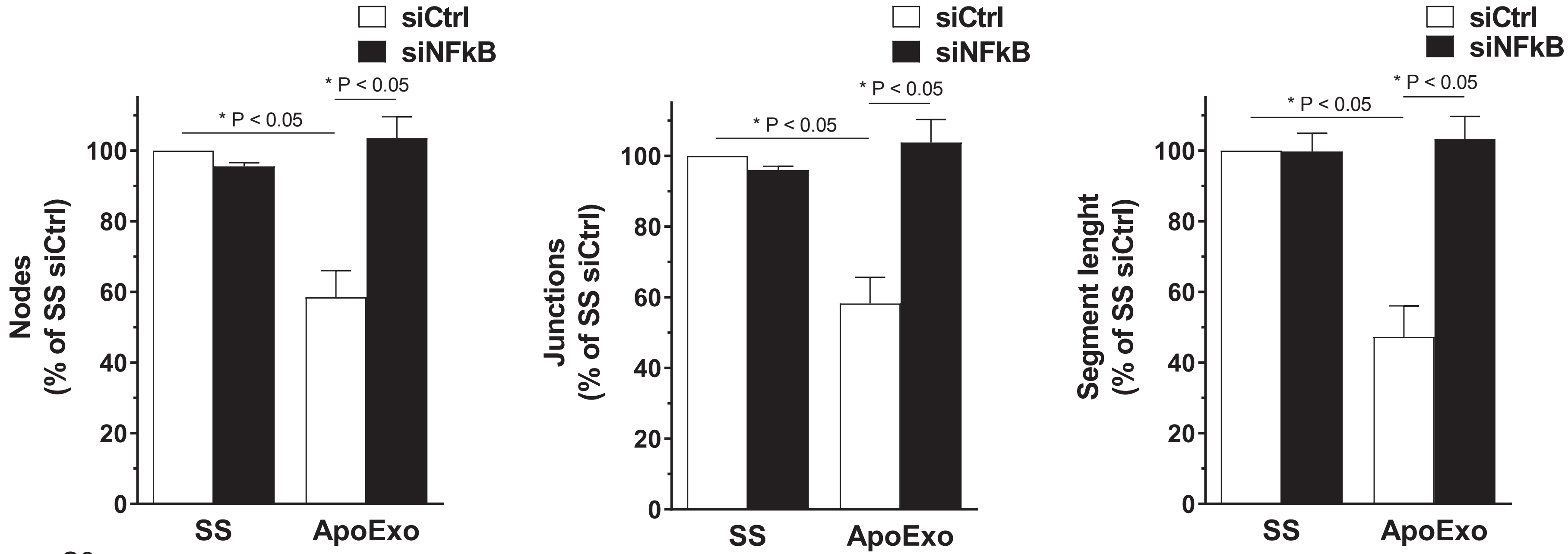
siCtrl
siNFkB
* P < 0.05
* P < 0.05
Nodes
(% of SS siCtrl)
100
80
60
40
20
0
SS
ApoExo
siCtrl
siNFkB
* P < 0.05
* P < 0.05
Junctions
(% of SS siCtrl)
100
80
60
40
20
0
SS
ApoExo
siCtrl
siNFkB
* P < 0.05
* P < 0.05
Segment lenght
(% of SS siCtrl)
100
80
60
40
20
0
SS
ApoExo

Figure S8

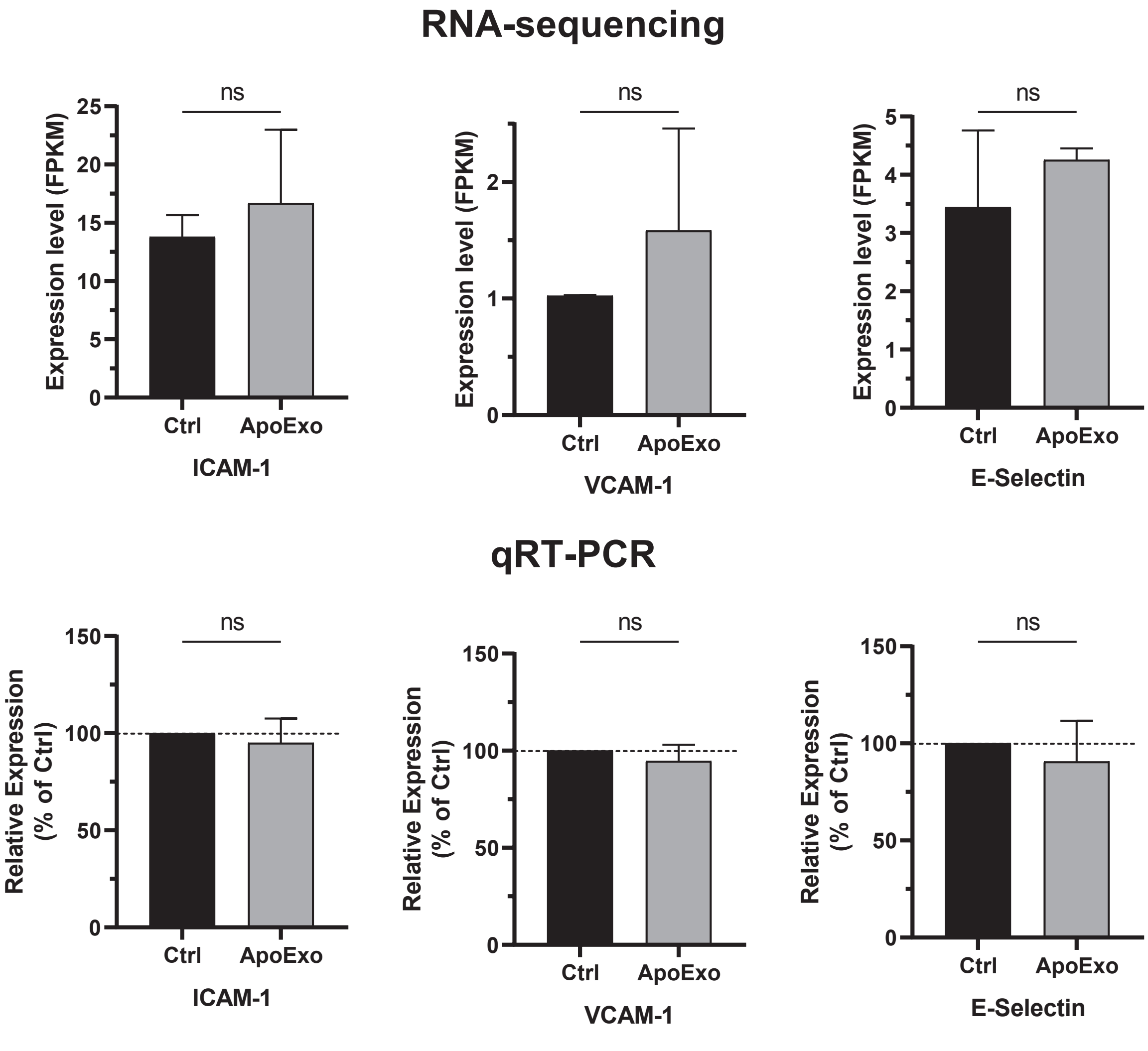
RNA-sequencing
ns
Expression level (FPKM)
25
20
15
10
5
0
Ctrl
ApoExo
ICAM-1
2
1
0
VCAM-1
4
3
E-Selectin
qRT-PCR
Relative Expression
(% of Ctrl)
150
100
50

Figure S9

A

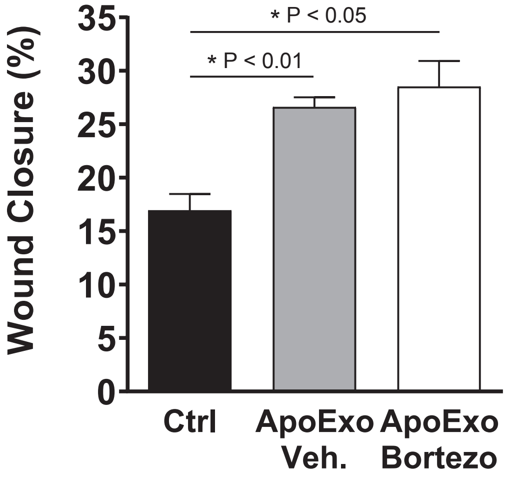

B

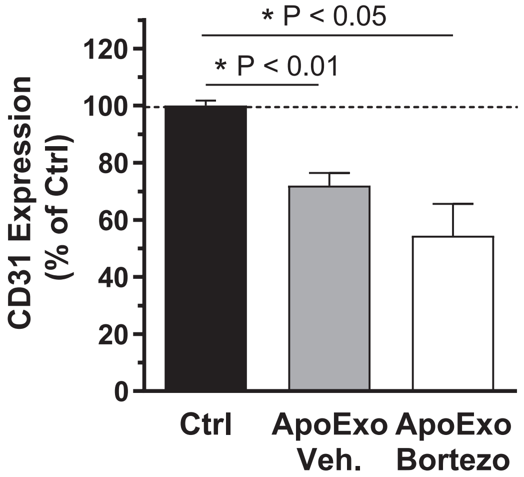

Figure S10

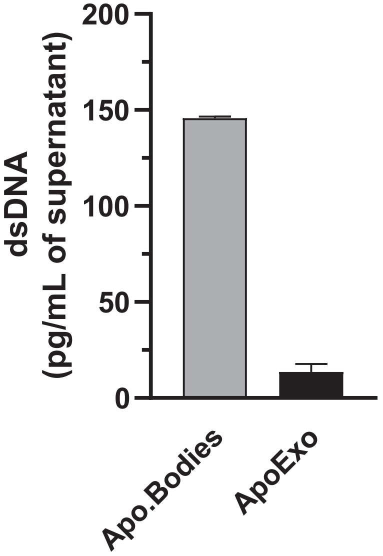

Figure S11

Fig. 4B

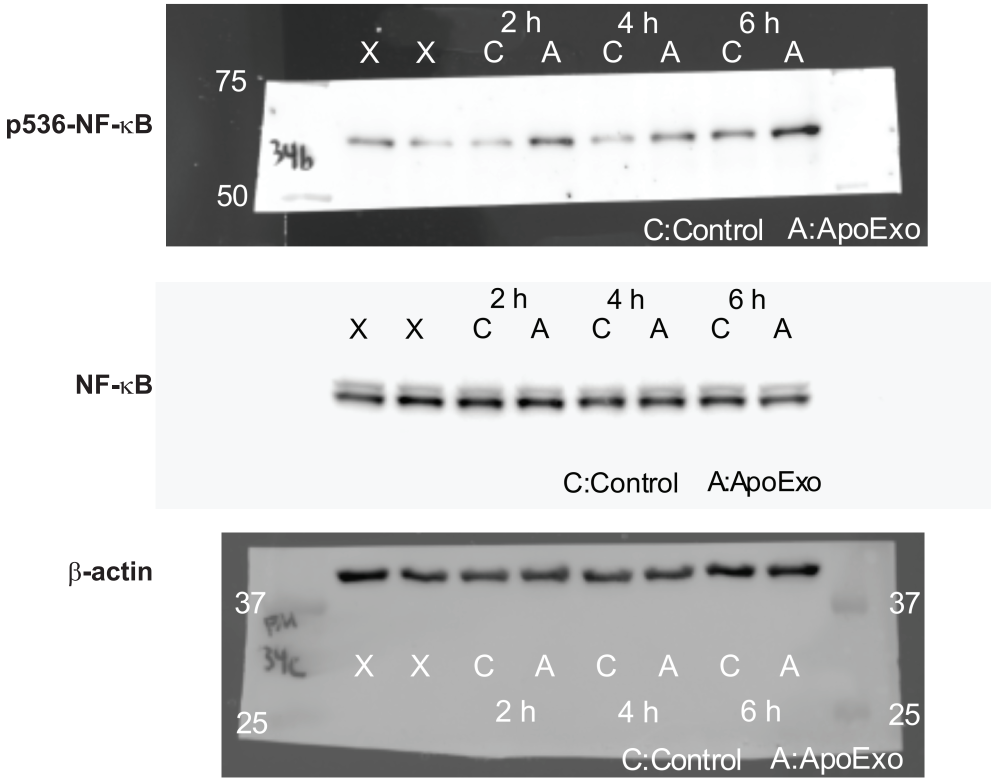

Fig. 4C

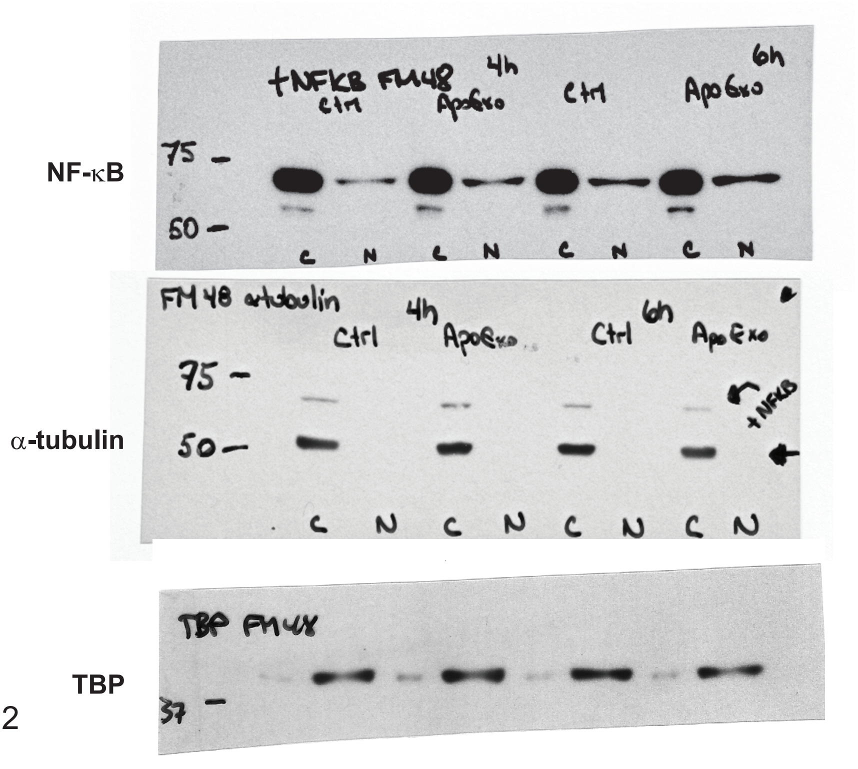

Figure S12

Fig. 5A

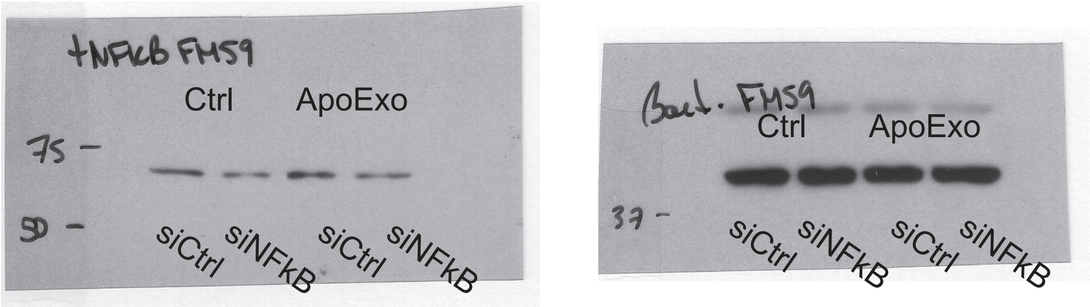

Fig. 5B

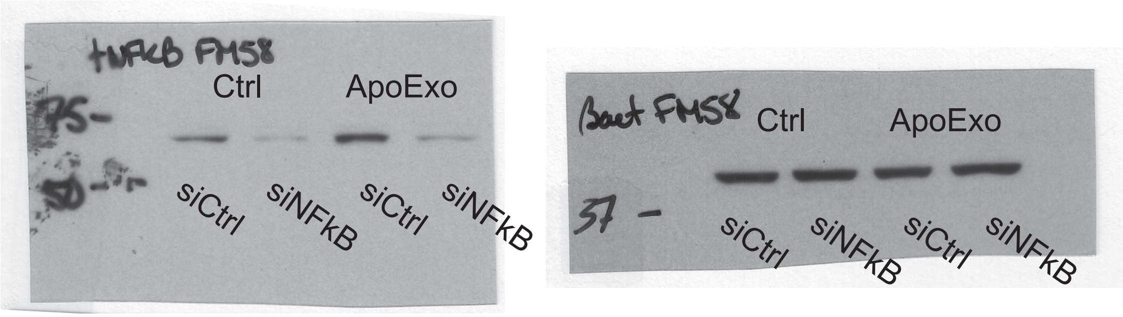

Fig. 5C

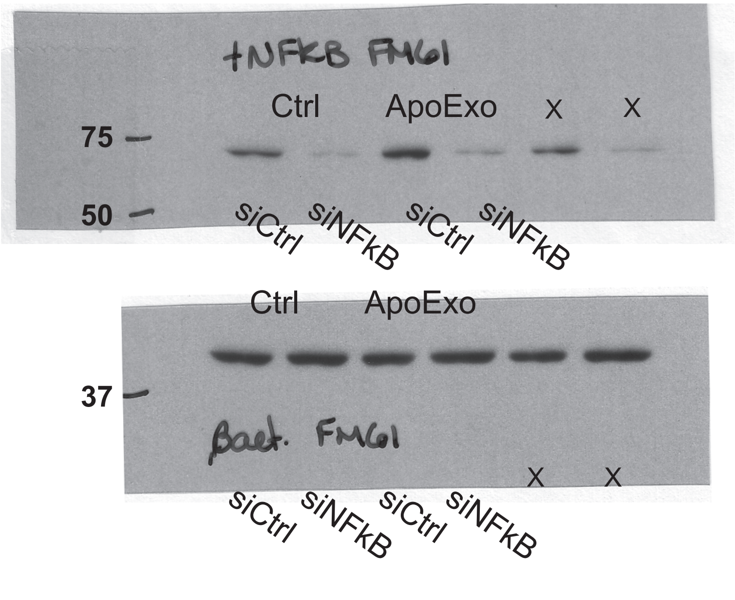

Fig. 5D

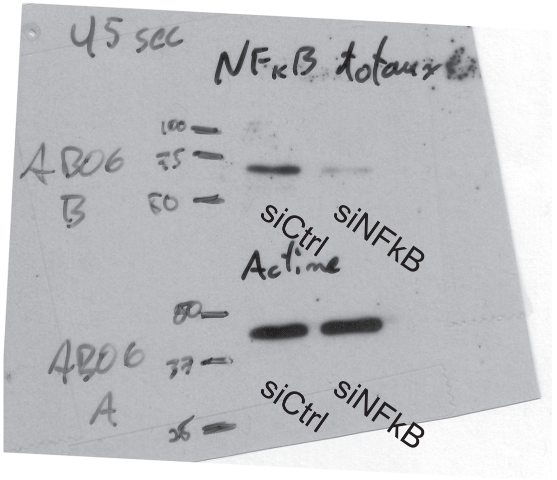

Figure S13

Cleaved caspase-3

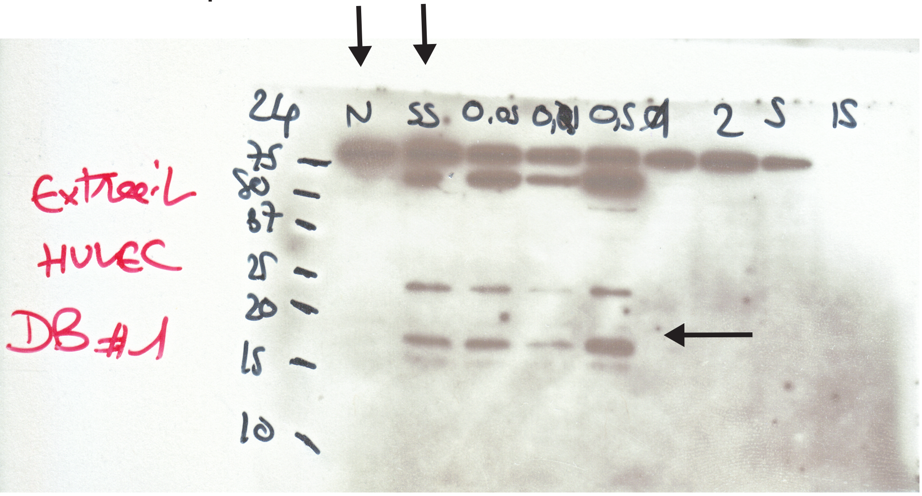

B-actin

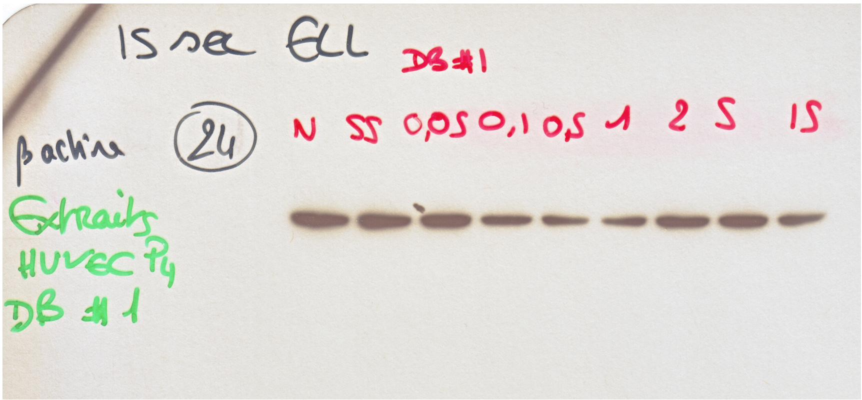

Figure S14

GM130

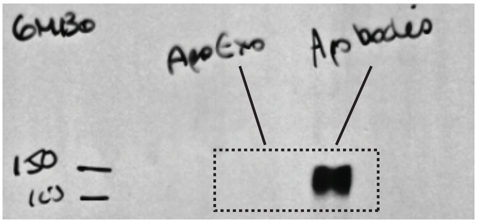

Synthenin

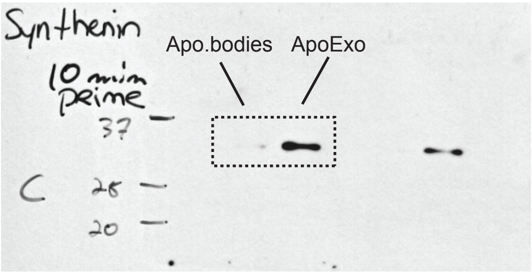

LAMP2

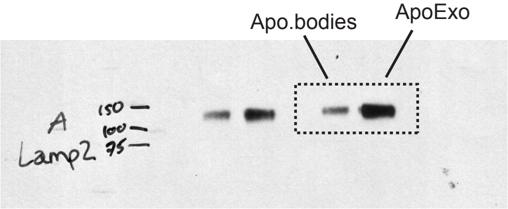

20S Proteasome α3

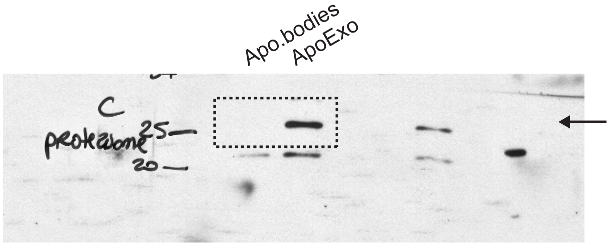

CD63

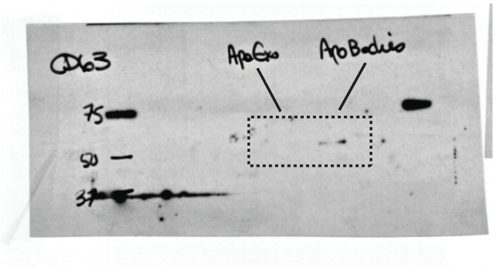

LG3

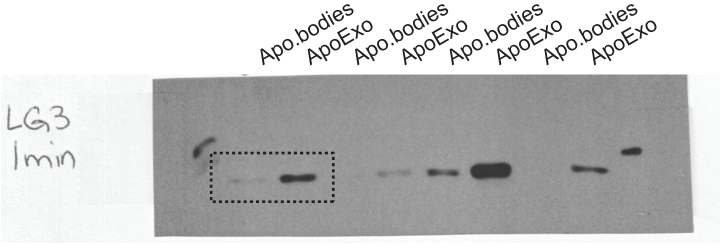

Tubulin

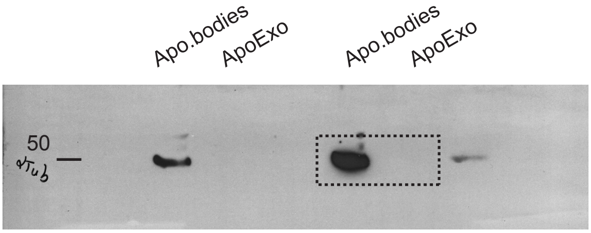

TCTP

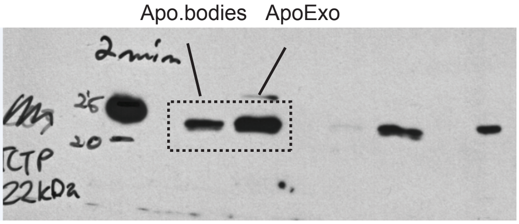

Figure S15

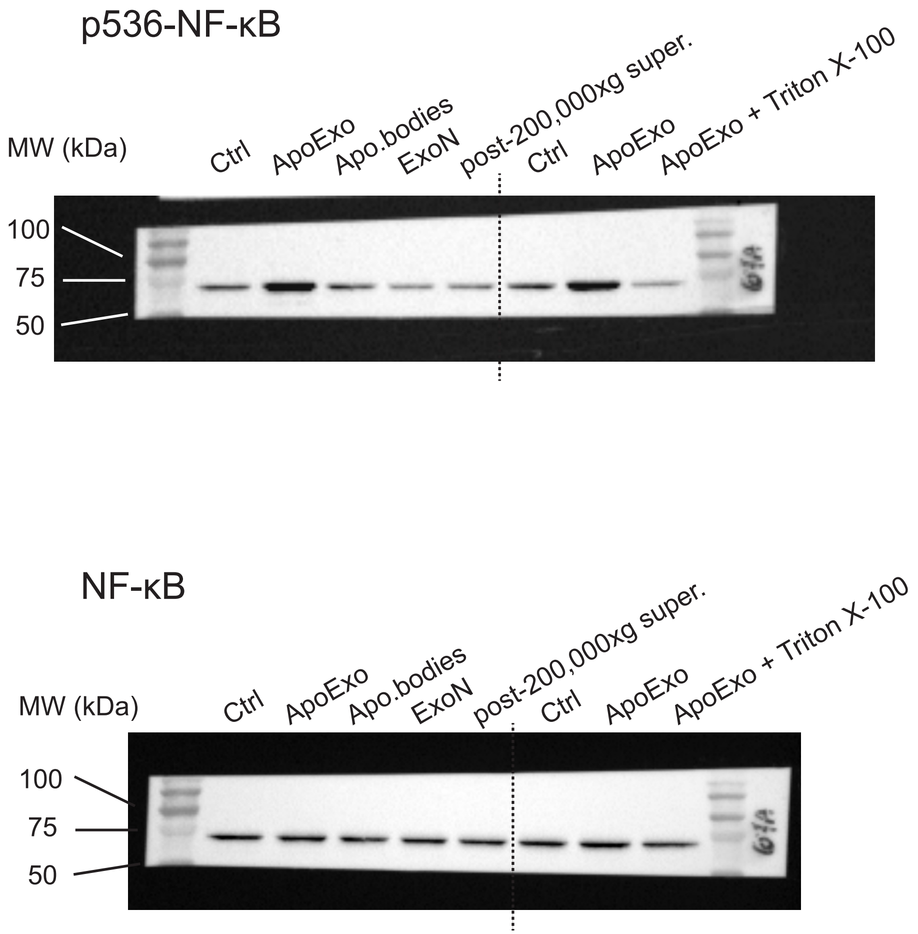
p536-NF-κB
MW (kDa)
Ctrl
ApoExo
Apo.bodies
ExoN
post-200,000xg super.
Ctrl
ApoExo
ApoExo + Triton X-100
100
75
50
NF-κB
MW (kDa)
Ctrl
ApoExo
Apo.bodies
ExoN
post-200,000xg super.
Ctrl
ApoExo
ApoExo + Triton X-100
100
75
50

Figure S16
